# Functionally important amino acid residues in the transient receptor potential vanilloid 1 (TRPV1) ion channel – an overview of the current mutational data

**DOI:** 10.1186/1744-8069-9-30

**Published:** 2013-06-22

**Authors:** Zoltán Winter, Andrea Buhala, Ferenc Ötvös, Katalin Jósvay, Csaba Vizler, György Dombi, Gerda Szakonyi, Zoltán Oláh

**Affiliations:** 1 Institute of Pharmaceutical Analysis, Faculty of Pharmacy, University of Szeged, Szeged, Hungary; 2 Institute of Biochemistry, Biological Research Centre of the Hungarian Academy of Sciences, Szeged, Hungary; 3 Acheuron Hungary Ltd, Szeged, Hungary

**Keywords:** TRPV1, Vanilloid, Amino acid, Mutants, Molecular modelling, Structure

## Abstract

This review aims to create an overview of the currently available results of site-directed mutagenesis studies on transient receptor potential vanilloid type 1 (TRPV1) receptor. Systematization of the vast number of data on the functionally important amino acid mutations of TRPV1 may provide a clearer picture of this field, and may promote a better understanding of the relationship between the structure and function of TRPV1. The review summarizes information on 112 unique mutated sites along the TRPV1, exchanged to multiple different residues in many cases. These mutations influence the effect or binding of different agonists, antagonists, and channel blockers, alter the responsiveness to heat, acid, and voltage dependence, affect the channel pore characteristics, and influence the regulation of the receptor function by phosphorylation, glycosylation, calmodulin, PIP2, ATP, and lipid binding. The main goal of this paper is to publish the above mentioned data in a form that facilitates *in silico* molecular modelling of the receptor by promoting easier establishment of boundary conditions. The better understanding of the structure-function relationship of TRPV1 may promote discovery of new, promising, more effective and safe drugs for treatment of neurogenic inflammation and pain-related diseases and may offer new opportunities for therapeutic interventions.

## Introduction

This review aims to create an overview of the currently available results of site-directed mutagenesis studies on transient receptor potential vanilloid type 1 (TRPV1) receptor. Systematization of the vast number of data on the functionally important amino acid mutations of TRPV1 may provide a clearer picture of this field, and may promote a better understanding of the relationship between the structure and function of TRPV1.

The first few sections provide a brief introduction of the transient receptor potential family and the TRPV1 ion channel. The following sections compile the positions crucial for the different channel functions and the amino acid changes of these 112 unique mutated sites of TRPV1. The information collected in this form may serve as a powerful tool for *in silico* molecular modelling by facilitating the establishment of boundary conditions.

All mutations and positions discussed in this paper generally refer to the rat ortholog (rTRPV1) of the TRPV1. All results relating to other species will be highlighted in the text.

### Transient receptor potential channels

The first TRP channel was discovered in 1969 by Cosens and Manning, who isolated a mutant photoreceptor from *Drosophila melanogaster*, which caused the specimen to become temporally blind upon exposure to bright light [1,2]. TRP channels are one of the largest families of ion channels. The mammalian TRP superfamily consisting of 28 TRP cation channels can be subdivided into six subfamilies: the TRPC (“Canonical”), TRPV (“Vanilloid”), TRPM (“Melastatin”), TRPP (“Polycystin”), TRPML (“MucoLipin”) and the TRPA (“Ankyrin”) groups [3,4].

All TRP channels are tetramers assembled with fourfold symmetry from the individual subunits containing six putative transmembrane segments (TMs). The region between the fifth and the sixth TMs, including a putative pore loop, forms the ion permeation pathway [5]. The amino and carboxyl (N- and C-) termini are located intracellularly and vary vastly in length and amino acid sequence. These cytoplasmic regions contain diverse well-recognized domains and motifs that are likely to be involved in channel assembly, activation and regulation through protein-protein and/or protein-ligand interactions. Most TRP channels are cation-selective, and some are highly selective for Ca^2+^ or Mg^2+^[4]. In accordance with their amino acid sequence diversity, TRP channels exhibit various activation and modulatory mechanisms, such as those involving in the stimulation by G-protein-coupled receptors, extracellular and intracellular ligands (including H^+^, Ca^2+^ and Mg^2+^), phosphatidylinositol-4,5-bisphosphate (PIP2), temperature, and mechanical stretch [6].

The accumulating evidence that TRP channels are important components of several human diseases has created a huge interest for these channels as novel potential drug targets. Comprehensive reviews have been published on the modulation and potential pharmaceutical application of TRP channels [7-11].

### TRPV subfamily

The TRPV family includes six mammalian ion channels, TRPV1-TRPV6, with a large C- and an even larger N-terminal cytoplasmatic domain containing ankyrin repeat domains (ARD) [12,13]. Ankyrin repeats, the 33-residue sequence motifs, are essential in channel function, ATP, PIP2, and calmodulin (CaM) binding [14] and protein-protein interactions [15,16]. They are present in many proteins, with functions including signalling, cytoskeleton integrity, transcription and cellular localization [17,18].

As polymodal thermo- and chemosensitive channels, TRPV1-TRPV4 are non-selective for cations and modestly permeable to Ca^2+^. In contrast, TRPV5 and TRPV6 are the only highly Ca^2+^-selective channels in the TRP family, and both channels are tightly regulated by the intracellular Ca^2+^ concentration [10,19,20].

TRPV channels can be activated through a variety of mechanisms. TRPV1-TRPV4 can act as thermosensors at a molecular level. Interestingly, each of these channels has a different thermal threshold for activation. When expressed in transfected permanent cell lines (HEK293 and CHO cells) and in frog oocytes [4,21,22], TRPV2, TRPV1, TRPV3 and TRPV4 are activated at 52°C , 43°C, 33°C, and below 33°C, respectively. The TRPV family members, except for TRPV1, are insensitive to vanilloid compounds [4]. TRPV3 can be activated by essential oils from clove (eugenol), thyme (thymol) and oregano (carvacrol) [23]. Like TRPV1, TRPV3 is also activated by camphor [24]. TRPV4 can be activated by cell swelling caused by 5',6'-epoxyeicosatrienoic acid [25]. TRPV5 and TRPV6, originally named ECaC [26] and CAT1 [27], are Ca^2+^ entry channels responsible for Ca^2+^ absorption in the kidney and intestine, respectively.

### TRPV1

In 1997, the breakthrough work of Michael Caterina and colleagues in the field of somatic sensory biology and pain research led to the cloning of the vanilloid (capsaicin) receptor, TRPV1 [28]. The cloning of TRPV1 proved to be a significant step in the understanding of molecular mechanisms that underlie the transduction of noxious thermal and chemical stimuli by sensory neurones [9].

#### Characteristics of TRPV1

**Figure 1:**
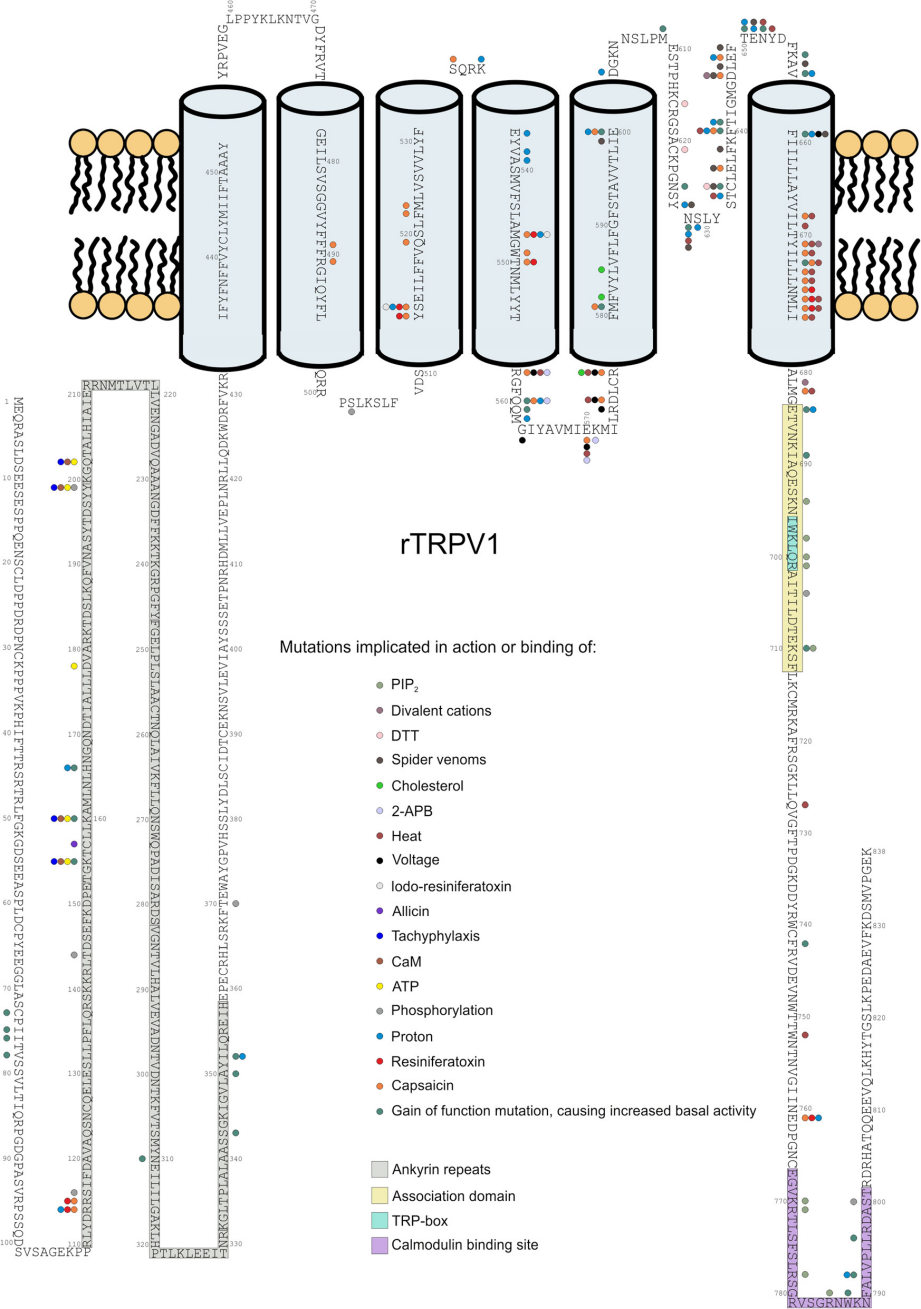
**Summary of the mutated sites of rTRPV1 The figure summarizes the effect of 112 unique mutated sites along the rTRPV1 on the function of the channel.** Coloured circles indicate the involvement of the given residue in the corresponding process. Coloured boxes show the putative location of some structural elements on the rTRPV1 sequence. Sequence and region information: TRPV1_RAT (O35433) (http://www.uniprot.org).

As a TRPV subfamily member, TRPV1 can be characterized by some key properties that are common among the members of the family. It is built from four individual subunits containing six TMs [29]. TRPV1 has a pore-forming hydrophobic stretch between TM5 and TM6 and is believed to exist as a homo- or heteromeric complex form [28-30] (Figure 1).

The TRPV1 receptor is a non-selective ligand-gated cation channel with a ninefold higher permeability for Ca^2+^ than for Na^+^. It is an integrator of a wide variety of exogenous and endogenous physical and chemical stimuli, including capsaicin (CAPS), noxious heat (>43°C) and protons (pH<5.2). Strictly speaking, CAPS and its biological analogs isolated from plants and animals are essentially sensitizers, because they act by lowering the thermal “physiological” activation threshold of TRPV1. Nevertheless, because these compounds bind directly to TRPV1, they are conveniently considered as direct activators, in contrast to compounds that do not bind TRPV1 and affect its functioning indirectly, which are referred to as sensitizers [31]. Besides CAPS, many complex amphiphilic molecules have been shown to activate or inhibit TRPV1. These include polyring compounds such as resiniferatoxin (RTX), a highly irritant diterpene related to the phorbol esters [32] which is almost 20-fold more potent than CAPS [33]. Species-specific differences in RTX potency have also been described, for example, RTX is a more potent agonist on rTRPV1 than its human ortholog (hTRPV1) [34,35]. Further molecules with similar structures, such as quinazolinone [36], evodiamine [37] and 17-β-estradiol [38], as well as molecules with long acyl and amide chains, such as anandamide [39-45], olvanil and omega-3 polyunsaturated fatty acids can also activate the channel [46]. Other natural TRPV1 agonists are 12-hydroperoxyeicosatetraenoic acid (12-HPETE) and N-arachidonoyl dopamine (NADA) [42,47-49]. Piperine from black pepper, eugenol from cloves and zingerone from ginger have also been shown to activate TRPV1 [50,51]. Additionally, gingerols, present in raw ginger, and shogaols, which are dehydration products of gingerols present in steamed ginger, both of which possess a vanillyl moiety, also activate TRPV1 [35,45,52,53]. Since both CAPS and its analogues are lipophilic, they are able to cross the cell membrane and act on binding sites present on the intracellular surface of TRPV1 [54]. TRPV1 can also be activated by 2-aminoethoxydiphenyl borate (2-APB). 2-APB inhibits both IP3 receptors and the majority of TRP channels, though at higher concentrations it activates TRPV1, TRPV2 and TRPV3 [11,55]. There has been debates about whether TRPV1 can [56-59] or can not [60-63] be activated by AITC. Stimuli are detected and transduced through opening of the ion channel, which results in the entry of cations such as Ca^2+^ and Na^+^ to the neurone [28]. Due to its uniqueness in its diverse operational features, which differ from the classical, more restrained proteins of voltage-gated and ligand-gated channels, as well as from the G-protein-coupled receptors, TRPV1 was dubbed “multisteric nocisensor” in the recent review paper by Szolcsányi and Sándor [64].

The inorganic dye Ruthenium Red (RuRed), is used in histology to stain aldehyde fixed mucopolysaccharides. Long before the cloning of TRPV1 RuRed was demonstrated to be able to inhibit CAPS-induced responses in sensory neurones [65]. Later it was shown that RuRed is a non-competitive antagonist not only for TRPV1 but for most other TRP channels functioning as a pore blocker [31]. The first competitive antagonist identified for TRPV1 was the CAPS analogue capsazepine (CapZ). CapZ is a relative potent antagonist on hTRPV1 but demonstrated much less potency on rTRPV1 [66,67], illustrating the differences between species that is common for many TRPV1 antagonists. Since the discovery of CapZ the number of TRPV1 patents has exceeded 1,000. Most of these antagonists show high affinity to TRPV1 and are competitive antagonists binding to the same site as CAPS and many of them have also demonstrated *in vivo* effects in various pain models [68]. The halogenated version of RTX, iodoresiniferatoxin (I-RTX) has been identified as a high-affinity antagonist of the TRPV1 channel and similarly to RTX, it also exert different potency on the hTRPV1 and rTRPV1 [69]. R4W2, a small positively charged peptide was identified to be non-competitive antagonist blocker of recombinant TRPV1 channels expressed in *Xenopus* oocytes [70], blocking CAPS-operated ionic currents with micromolar efficacy in a weakly voltage-dependent manner. R4W2 was later found to be competitive antagonist of TRPV1 also in primary cultures of adult rat dorsal root ganglion neurons (DRGs) [71].

Besides its involvement in pain sensation, TRPV1 displays a low level of activity at normal body temperature [72,73]. Constitutive activity of TRPV1 is essential for regulation of body temperature, evidenced by high fever as a adverse side effect of many TRPV1 blockers during clinical trials for their efficacy in management or prophylaxis of pain [74,75]. Moreover, at room temperature (24°C) and pH 7.3, TRPV1 behaves as a voltage-gated outwardly rectifying channel, since it can be activated, in the absence of any agonist, by depolarizing voltages (> +60 mV) [76].

One striking feature of TRPV1 is that the receptor can be sensitized and desensitized. This fact suggests that the TRPV1 function is subject to extensive modulation, which has significant implications for the involvement of TRPV1 in physiological and pathophysiological conditions. Some inflammatory mediators in damaged tissues including growth factors, neurotransmitters, peptides or small proteins, lipids, chemokines and cytokines sensitize TRPV1 to its agonists [77]. Even in concentrations that fail to activate a current, CAPS can sensitize TRPV1 channels to protons and heat. Similarly, protons can sensitize TRPV1 channels to CAPS and heat [78,79]. The elevation of temperature or local acidity can in principle augment the efficacies of partial agonists, transforming them from weakly or non-pain-producing ligands into noxious chemicals [80,81]. Whereas protons sensitize TRPV1 directly, most of the mediators work through receptor pathways, which include receptor tyrosine kinases and G-protein-coupled receptors. It has been reported that phosphorylation by protein kinase A (PKA) [82-84] and protein kinase C (PKC) can sensitize TRPV1 to CAPS, protons or heat [47,78,85-88]. The phosphorylation of TRPV1 by PKC acts to potentiate CAPS- or proton-evoked responses and reduces the temperature threshold for TRPV1 activation. Others have suggested that isoforms of PKCα [89] or PKCμ [90] are responsible for the effects described above. Protein kinase D/PKCμ is a member of the protein kinase D serine/threonine kinase family that exhibits structural, enzymological and regulatory features distinct from those of the PKCs, with which they are related. TRPV1 can also be phosphorylated by Ca^2+^ CaM-dependent kinase II (CaMKII) [91], or Src kinase [92], whilst the phosphatase calcineurin produces desensitization of the TRPV1 receptor [93]. TRPV1 undergoes two types of desensitization on activation by CAPS or protons: acute (short-term) desensitization and tachyphylaxis or loss of sensitivity to repeated stimulations [14,94]. Physiologically, TRPV1 desensitization can lead to the adaptation of peripheral neurones to pain perception. The regulatory lipid PIP2 is a putative intracellular modulator of TRPV1, although there is some debate as to whether it sensitizes or desensitizes the channel. Mutations in a C-terminal cytosolic region of TRPV1 indicate an inhibitory role for PIP2 [95]. However, others have found that PIP2 sensitizes TRPV1 and that depletion leads to desensitization [14,96-99]. Another important membrane lipid in terms of TRPV1 activity is cholesterol. Cholesterol is a major component of plasma membranes and is enriched in lipid rafts. It has been shown to modify the function of many classes of ion channels [100]. Cholesterol can modify channel activity indirectly by altering physical properties of the surrounding lipid bilayer, and the highly ordered lipid rafts can serve as organizing centres for many signalling processes [101]. In recent years compelling evidence has emerged of a specific interaction between cholesterol and several channels [100-103]. A supporting role of sphingomyelin and gangliosides was also demonstrated [104]. Sántha et al. demonstrated that inhibition of neuronal ganglioside synthesis by inhibition of glucosylceramide synthase reversibly decreased the CAPS-induced activation and TRPV1 expression of cultured dorsal root ganglion neurons, apparently leaving other markers of nociceptive neurons, such as CGRP and IB4, unaffected [105]. Intracellular ATP can also sensitize TRPV1.

TRPV1 binds and is modulated by Ca^2+^/CaM, a ubiquitous Ca^2+^ sensor [106,107]. An increase in intracellular Ca^2+^ concentration causes TRPV1 desensitization, and CaM plays a role in mediating this effect [106-108]. CaM interacts *in vitro* with isolated peptides from the TRPV1 N-terminal region in a Ca^2+^-dependent manner [107], and also binds to the TRPV1 C-terminal region in a Ca^2+^-independent manner [106]. The response of TRPV1 to heat can be modified by tyrosine kinases or G-protein-coupled receptors. Channel activation can occur even at normal body temperatures [78,109,110]. Reducing agents such as dithiothreitol (DTT) strongly increase the thermally induced activity of the TRPV1 channel [111]. Moreover, the oxidizing agents diamide and chloramine-T also facilitate thermally induced TRPV1-mediated currents [112]. Alkylating agents, such as N-ethylmaleimide also strongly and irreversibly affect heat-evoked responses from TRPV1, lowering the thermal activation threshold in a DTT-dependent manner [112]. It follows from these data that TRPV1 is targeted by redox-active substances that directly modulate the channel activity, and that channel potentiation may occur under altered redox states in a tissue, e.g., during ischaemia and/or inflammation, presumably leading to hyperalgesia.

Inflammatory agents such as bradykinin, serotonin, histamine, or prostaglandins can further stimulate TRPV1 activity, either by PKC-dependent pathways [110,113,114], by releasing the channel from PIP2-dependent inhibition [99,115], by a PKA-mediated recovery from inactivation [116,], or by the formation of 12-HPETE [78,117].

#### Vitally important functions of TRPV1

The role of the TRP channels in pain and neurogenic inflammation have been very well covered by previous authors (e.g. [118-121]) reflecting the enormity of the role that these channels play in sensory nerve function at both a central and peripheral level [122]. Thus TRPV1 plays a key role in the development of the burning pain sensation associated with acute exposure to heat or CAPS, and with inflammation in peripheral tissues [28,91]. The receptor seems to play important role in certain chronic pain conditions, such as neuropathic pain, osteoarthritis, bone cancer pain, inflammatory bowel disease and migraine [9,123,124]. Its role in the central nervous system (CNS) is known to involve pain processing and modulation, neurogenesis [125] and thermoregulation [126], amongst others, but is currently less well understood.

Jancsó and Wollemann [127] have reported that CAPS stimulates adenylate cyclase activity in the rat cerebral cortex *in vitro*. Furthermore, direct injection of CAPS into the preoptic area of the anterior hypothalamus [126] or i.c.v. region [128] of the rat brain causes hypothermia, suggesting a role for this channel in thermoregulation. Similarly, systemic administration of TRPV1 antagonists such as AMG517 [73], AMG0347 [129] and A-425619 [130] causes an increase in body temperature within approximately 1 h of treatment [122]. However, antagonist-induced hyperthermia may not be mediated by hypothalamic TRPV1 as peripherally-restricted antagonists still have the capacity to cause an increase in body temperature [131].

Within the periphery, recent evidence has located TRPV1 on a variety of non-neuronal tissues (for more details see Table two in the paper of Fernandes et al. [122]). Although TRPV1 channel expression has been shown in a wide variety of tissues, evidence of functionality has not yet been demonstrated for all of these.

One of the first cell types in which functionality was first identified is epidermal keratinocytes. Inoue et al. [132] demonstrated that both CAPS and acidification produced elevations in the intracellular calcium concentration in cultured human epidermal keratinocytes that could be inhibited by the TRPV1 antagonist, CapZ [122]. Similarly, treatment of human skin fibroblasts with CAPS induced significant changes in the membrane current and the intracellular calcium level that were antagonized by CapZ [133]. It is therefore clear that TRPV1 is expressed and functional away from sensory nerves.

Physiological or pathophysiological effects of non-neuronal TRPV1 have been implicated in inflammation, infection and immunity, the cardiovascular system and in conditions such as obesity [122].

A physiological/pathophysiological role for non-neuronal TRPV1 is perhaps nowhere more apparent than in the case of inflammation, infection and immunity. Although the effects of these TRPV1 channels are non-neuronal, it is evident that they may well impact indirectly upon pain and/or neurogenic inflammation [122]. Keratinocytes functionally express TRPV1. These cells play an important role in maintaining the integrity of the immune response in skin as well as stimulating cutaneous inflammation via prostanoid and cytokine release [134]. TRPV1 activation by CAPS causes an increase in COX2 expression in human keratinocytes with a concomitant increase in PGE_2_ levels *in vitro*[135]. An increase in IL-8 is also observed [135] Stimulation of inflammatory mediator release by TRPV1 agonists from keratinocytes could well have a significant effect upon sensory nerves that have a high density in skin [122].

However, there is accumulating evidence that TRPV1 has functional roles away from sensory nerve activity [122]. As well as keratinocytes, peripheral blood mononuclear cells (PBMCs) are also directly affected by TRPV1 activation. For example, PBMCs undergo apoptosis when stimulated with CAPS or RTX, an effect that is reversed by the TRPV1 antagonist, AM630 [136]. There are various other cells involved in immunity that respond to TRPV1 and TRPA1 activation including bone marrow-derived dendritic cells, where CAPS leads to dendritic cell maturation and an increase in antigen presentation [137].

TRPV1 is expressed, functional and active within cells relevant to inflammation, infection and immunity. It is clear from the studies that TRPV1 at least plays a paradoxical role in inflammation *in vivo*, for example, exacerbating inflammation in arthritis and yet in experimentally induced sepsis, TRPV1 null mice demonstrate elevated levels of pathological markers in comparison with wild type mice [123]. It cannot be ruled out that this is due to differing effects of neuronal and non-neuronal TRPV1 channels [122].

TRPV1 have been shown to control vascular responses either by the well-established neurogenic response that is mediated by sensory nerves [138] or via a direct effect on vascular tissue [139,140]. However, the non-neuronal mechanisms involved in mediating vasodilatation and oedema formation following TRPV1 activation *in vivo* are unclear. TRPV1 on endothelial cells has been shown to regulate the expression and secretion of endothelial cell-derived CGRP, which affords protective effects on endothelial cells [141]. Furthermore, CGRP is a potent vasodilator [142], and this CGRP may therefore impact upon blood pressure. Indeed, TRPV1 activation on sensory nerves also causes CGRP release, leading to a profound decrease in vascular tone [143]. On the other hand, TRPV1 expressed on vascular smooth muscle appears to cause vasoconstriction [140,144,145]. It has also been suggested that CAPS has biphasic effects on the vasculature: at lower concentrations, CAPS (up to 10 nM) evokes vasodilation in skin due to sensory nerve activation, whereas higher concentrations (0.1–1 mM) elicit substantial constrictions in skeletal muscle arterioles due to non-neuronal TRPV1 stimulation [140]. It is unclear whether this difference is due to receptor sensitivity or a difference in TRPV1 receptor density in the two tissues. A similar biphasic effect of CAPS has been demonstrated also in meningeal blood vessels [146]. TRPV1 may also play a role in vascular responses during chronic hypoxia where up-regulation of the TRPV1 gene and protein is observed [147]. Chronic hypoxia has been shown to enhance the ability of human pulmonary artery smooth muscle cells to proliferate and to increase resting levels of cytosolic calcium and capacitative calcium entry with both effects being inhibited in a dose-dependent manner by the TRPV1 antagonist, CapZ [147]. These results suggest that TRPV1 on smooth muscle may be a critical pathway or mediator in chronic hypoxia-induced vascular changes [122].

Obesity is one of the most significant health issues in western society due to the morbidity associated with this condition that is increasing in prevalence. Obesity is induced by the hypertrophy of adipocytes and the recruitment of new adipocytes from precursor cells. These processes are dependent on the regulation of adipocyte differentiation. CAPS has been shown to inhibit adipocyte differentiation *in vitro* by activation of AMP-activated protein kinase [148]. Furthermore, Hsu and Yen [149] have shown that treatment of preadipocytes with CAPS decreases the number of normal adipocytes and increases the number of early apoptotic and late apoptotic cells in a dose-dependent manner. Thus the overall effect of TRPV1 modulation in obesity is stark. Both animal [150] and human [151] data have indicated that the consumption of CAPS- or non-pungent capsiate-containing foods is correlated with a reduced incidence of obesity. Similarly, oral administration of CAPS alone also suppresses body fat accumulation in mice [151], and dietary CAPS can reduce obesity-induced insulin resistance and hepatic steatosis in mice fed a high fat diet [122,152].

In recent years, a role for TRPV1 in thermoregulation has also been identified which may, at least in part, be due to changes in thermogenesis (for review, see [72,153,154]). For many years, CAPS has been known to cause a centrally mediated hypothermia in mice [126]. In contrast, its intragastric administration enhances thermogenesis and heat diffusion [155]. Similarly, the jejunal administration of non-pungent CAPS analogues was shown to increase energy expenditure via direct activation of TRPV1 located on intestinal extrinsic nerves [156]. Interestingly, some TRPV1 antagonists cause hyperthermia, associated with increased thermogenesis [130] through a peripheral mechanism [131], whilst TRPV1 gene knock down does not affect body temperature in mice [157] and TRPV1 knockout mice exhibit a normal basal body temperature [122,129].

TRPV1 in the alimentary tract has also been implicated in metabolism, hair growth regulation [158], and the development of cancer [159]*.*

Consequently, control of the TRPV1 function may have the potential to provide exciting opportunities for therapeutic interventions. Its known functions in both health and disease have been continuously expanding promoting a better understanding of TRPV1.

#### TRPV1 expression

TRPV1 channels are mainly expressed on primary sensory neurones. They have been detected in key areas of the pain transduction pathway: on terminals of small- to medium-diameter nociceptors, such as peptidergic and non-peptidergic C fibres, some Aδ fibres [28,79]; in skin nerve endings, DRGs, nodose ganglia, trigeminal ganglia of the peripheral nervous system, as well as laminae I and II [79] of the dorsal horn of the spinal chord. Projections may also extend into laminae V and X [79]. Moreover TRPV1 has been found in different brain regions, such as in dopaminergic neurones of the substantia nigra, hippocampal pyramidal neurones, hypothalamic neurones, neurones in the locus coeruleus, and in various layers of the cortex [25,160,161]. The channel is present to a lesser extent in the hippocampus, cortex, olfactory bulb and cerebellum in the CNS [44,162]. However, more recently, the use of TRPV1 reporter mice has revolutionized the study of TRPV1 expression, and they would suggest that the expression of this receptor is minimal within a few discrete brain regions, most obviously in the vicinity of the caudal hypothalamus [122,145].

TRPV1 is additionally expressed in discrete spots in the plasma membrane and cytosol of different non-neuronal cells such as the endothelium, immune cells (lymphocytes, dendritic cells and mast cells), keratinocytes, smooth muscle cells and urothelium, thymocytes and macrophages, some of which are known to be involved in inflammation [159]. An increased level of TRPV1 expression has been demonstrated in the sensory fibres of patients with an inflamed oesophagus (gastro-oesophageal reflex disease) or an inflamed bowel (both ulcerative colitis and Crohn’s disease), or with chronic breast tenderness and pain [163]. There is likewise an increased TRPV1 expression in the sensory fibres of patients with rectal hypersensitivity and faecal urgency, and this increase is directly correlated with the degree of thermal and mechanical sensitivity [164].

## Functionally important amino acid sites and their mutations in the TRPV1

Emerging data of TRP channel research has elucidated roles for TRP channels in diverse therapeutic areas, and resulted in the identification of numerous potential drug targets beyond TRPV1. Success in finding a viable therapy targeting the TRPV1 channel depends on experimental studies aimed at obtaining detailed knowledge of the channel protein. TRPV1 pharmacology has relied heavily upon information derived from studies of site-directed mutants of the recombinant channel(s) by the identification of the most appropriate acting sites for potential drug candidates. Figure 1 indicates the mutated residues and the functional changes evoked by them, and also depicts the most important structural properties of TRPV1 (For high resolution figure, see Additional file 1). In order the easier comprehensibility and the more effective usability of the data, a comprehensive table summarizes the information of the mutants at the end of the article (see Table 1).

**Table 1:** Summary table of the mutants discussed in the article

**Residue**	**Mutated to**	**Role in the channel function / Impact of the mutation on the channel function**	**Refs**
C73	S	Its mutation caused gain of function and strong toxicity when expressed in *Saccharomyces cerevisiae.*	[177]
I75	F	Its mutation caused gain of function mutation and weaker toxicity when expressed in *Saccharomyces cerevisiae.*	[177]
I76	T	Its mutation caused gain of function mutation and weaker toxicity when expressed in *Saccharomyces cerevisiae.*	[177]
V78	G	Its mutation caused gain of function mutation and weaker toxicity when expressed in *Saccharomyces cerevisiae.*	[177]
R114	A, E, Δ	Its deletion or mutation abrogated vanilloid and proton activation and RTX binding, without effecting heat activation.	[16]
R115	D	Its mutation abrogated vanilloid activation and RTX binding, without effecting heat activation.	[16]
S116	A	The residue is phosphorylated by PKA and is involved in desensitization. It is also functional target for PKCμ. Its mutation abolished phosphorylation by PKCμ and enhanced the channel response to CAPS by PKCμ.	[85,90,176,210]
T144		Its phosphorylation by PKA causes sensitization of heat-evoked responses.	[78,83]
K155	A, E	Its mutation caused impaired TRPV1-ARD interaction with ATP, and impaired tachyphylaxis, even in the absence of ATP. The mutant channel did not interact with CaM, and was slightly more sensitive to CAPS than the wild type.	[14,177]
		Its mutation resulted in a constitutively active channel, and caused gain of function mutation and weaker toxicity when expressed in *Saccharomyces cerevisiae.*	
C157		The residue is covalently modified by allicin causing allicin activation of the channel.	[187]
K160	A, E	Its mutation caused impaired TRPV1-ARD interaction with ATP, and impaired tachyphylaxis, even in the absence of ATP. The mutant channel did not interact with CaM, and was slightly more sensitive to CAPS than the wild type.	[14,177]
		Its mutation resulted in a constitutively active channel, caused gain of function mutation and strong toxicity when expressed in *Saccharomyces cerevisiae.*	
H166	R	Its mutation caused gain of function mutation and weaker toxicity when expressed in *Saccharomyces cerevisiae.* Its mutation resulted in significant response to pH 6.4 (threshold concentration for proton-activation).	[177]
D178	N	Its mutation abolished the ATP-mediated upregulation of TRPV1.	[205]
Y199	A, F	When both sites were mutated, Y199/Q202 impaired the TRPV1-ARD interaction with ATP and ATP-mediated tachyphylaxis. The mutated channel was slightly more sensitive to CAPS than the wild type. Mutant channels formed a weaker complex with CaM than wild type but it still had a 1:1 stoichiometric ratio.	[14,211]
		Phosphorylation of hTRPV1 Y200 (Y199 in rTRPV1) by Src kinase increases the surface expression of TRPV1 and accounts for rapid sensitizing actions of NGF. When mutated, Src-dependent, NGF-induced Tyr phosphorylation was completely abolished.	
Q202	A	When both sites were mutated, Y199/Q202 impaired the TRPV1-ARD interaction with ATP and ATP-mediated tachyphylaxis. The mutated channel was slightly more sensitive to CAPS than the wild type. Mutant channels formed a weaker complex with CaM than wild type but it still had a 1:1 stoichiometric ratio.	[14]
N310	D	Its mutation caused gain of function and weaker toxicity when expressed in *Saccharomyces cerevisiae.*	[177]
S343	G, R,	Its mutation caused gain of function and strong/weak toxicity when expressed in *Saccharomyces cerevisiae.*	[177]
A350	T	Its mutation caused gain of function and strong toxicity when expressed in *Saccharomyces cerevisiae.*	[177]
I352	N, T	Its mutation caused gain of function mutation and weaker toxicity when expressed in *Saccharomyces cerevisiae.* Its mutation also caused significant response to pH 6.4 (threshold concentration for proton-activation).	[177]
T370		The residue is phosphorylated by PKA. It is involved in desensitization of the channel, and in the sensitization of heat-evoked TRPV1 responses when phosphorylated by PKA.	[78,83,85,176,210]
F489	Y	Mutation resulted in a rightward shift of the CAPS concentration of half-maximal activation.	[167]
R491	E, G,	Mutation caused reduction in CAPS sensitivity.	[168]
S502	A	Its phosphorylation by PKC potentiates CAPS, proton, and thermal responses, and that by PKA sensitizes the heat-evoked responses.	[78,83,85,91,174,209]
		It is a CaMKII phosphorylation site.	
		When associated with T704I, S502A was found to lose the ability to be activated by CAPS and to lose the ability of vanilloid binding.	
		Its mutation reduced PMA enhancement of CAPS-evoked currents, but had no effect on direct activation by PMA.	
Y511	A, C, F,	Its mutation abolished CAPS responses, and RTX binding yet leave activation by heat and protons intact.	[168-171,180]
S512	A, F, T, Y	Its mutation abolished CAPS responses and RTX binding, yet left activation by heat and protons intact. It is involved in I-RTX binding.	[168,169,173,193]
Q519	N	Its mutation ablated the vanilloid sensitivity.	[168]
F522	L	Its mutation ablated the vanilloid sensitivity.	[168]
M523	L	Its mutation ablated the vanilloid sensitivity.	[168]
S532	C	Its mutation ablated the vanilloid sensitivity.	[168]
K535	E	Its mutation ablated the vanilloid sensitivity, and affected proton responses.	[168,192]
E536	L, W	Its mutation ablated the vanilloid sensitivity, and affected proton responses.	[168,192]
V538	A, G, I, L, T	Its mutation ablated proton activation, but not the proton potentiation.	[192]
A539	P	Its mutation affected proton responses.	[192]
M547	A, I, L, Q	The residue is involved in RTX binding, CAPS sensitivity, I-RTX sensitivity and proton sensitivity.	[168,170,171,173,180]
W549		It is involved in vanilloid binding.	[171,182]
T550	A, C, I, S, Y, Δ,	It is an important molecular determinant in vanilloid sensitivity. Participates in Caps and RTX binding.	[168,170,171,180]
		Its deletion reduced CAPS sensitivity of the channel.	
R557	A, E, K, L	It is involved in CAPS potentiation of heat-induced currents and in the transduction of the CAPS-binding signal to the opening of the pore.	[179]
		It is also important in deactivation gating process, 2-APB activation and for voltage-dependent gating.	
		It contributes to the voltage modulation of the CAPS-induced currents and the CAPS potentiation of the heat-induced currents.	
Q560	H, R	It is involved in the transduction of the CAPS-binding signal to the opening of the pore.	[177,179]
		It is also important in deactivation gating process and for 2-APB activation.	
		Its mutation caused significant response to pH 6.4 (threshold concentration for proton-activation).	
		Its mutation caused gain of function of the channel and weaker toxicity when expressed in *Saccharomyces cerevisiae.*	
Q561	H, R	Its mutation caused gain of function of the channel and strong toxicity when expressed in *Saccharomyces cerevisiae.*	[177]
M562	D	Its mutation caused significant response to pH 6.4 (threshold concentration for proton-activation).	[177]
G563	S	The residue is involved in the voltage gating of the channel.	[179]
E570	A, L, Q, R	The residue contributes to the voltage modulation of the CAPS-induced currents and the CAPS potentiation of the heat-induced currents.	[179]
		It is involved in the transduction of the CAPS-binding signal to the opening of the pore and in the 2-APB activation of the channel.	
		It is also a relevant heat-sensing factor.	
K571	E	The residue is a specific binding site for 2-APB.	[172,179,191,196]
		It is involved in voltage sensing and in TRPV1-lipid interactions.	
R575	A	The residue is involved in voltage sensing and in TRPV1-lipid interactions.	[172,191,196]
D576	N, R	The residue is involved in the voltage-dependent gating of TRPV1, and contributes to the voltage modulation of the CAPS-induced currents and the CAPS potentiation of the heat-induced currents.	[179]
		It contributes to the transduction of the CAPS-binding signal to the opening of the pore.	
R579	A, D, E	The residue contributes to the voltage modulation of the CAPS-induced currents and to the CAPS potentiation of heat-induced currents. It is involved in the voltage sensing and in TRPV1-lipid interactions. Its mutation decreased the cholesterol response of the channel.	[172,179,191,196,208]
M581	T	The residue contributes to the transduction of the CAPS-binding signal to the opening of the pore, and to the deactivation gating process. It is involved in voltage gating.	[177,179]
		Its mutation caused gain of function of the channel and strong toxicity when expressed in *Saccharomyces cerevisiae.*	
F582	Q	Its mutation decreased the cholesterol response of the channel.	[208]
L585	I	Its mutation abolished the cholesterol response of the channel.	[208]
I599	A	Its mutation caused reduced DkTx responses.	[207]
E600	A, D, H, K, Q, S, V	The residue is involved in the proton potentiation, but not in proton activation.	[43,167]
		Its mutation to neutral or positive residues potentiates responses to CAPS or heat, and introduction of a residue with lower pKa decreased the channel sensitivity to CAPS or heat.	
D601	N	Its mutation reduced the proton-activated currents significantly, without altering the heat- or CAPS-evoked responses, and without eliminating the ability of protons to potentiate the responses to these stimuli.	[43]
M609	T, V	Its mutation caused gain of function of the channel and strong/weak toxicity when expressed in *Saccharomyces cerevisiae.*	[177]
C616	G	The residue is involved in DTT interaction.	[78,112,207]
C621	G	The residue is responsible for the extracellular modulation of TRPV1 by reducing agents. It is involved in DTT interaction. Its mutation and when associated with C616G and C634G significantly reduced DDT potentiation without having any effect on the CAPS, heat or voltage gating of the channel.	[78,112,207]
N625	D	Its mutation caused toxicity when expressed in *Saccharomyces cerevisiae.*	[177]
Y627	A, W	The residue contributes to, but do not play a pivotal role in the proton activation. Its mutation enhanced the sensitivity to the acylpolyamine toxins AG489 and AG505.	[192,200]
N628	D, K, R, W	The residue plays essential roles in the heat response without affecting the CAPS responses or the desensitization of the channel. It is involved in proton-induced potentiation. Its mutation reduced the heat responses in amplitude and shifted them to higher temperatures, dramatically decreased the sensitivity to the acylpolyamine toxins AG489 and AG505 and caused toxicity when expressed in *Saccharomyces cerevisiae.*	[167,177,192,200,213]
S629	A	The residue contributes to, but do not play a pivotal role in the proton activation.	[192]
T633	A, S, V	Its mutation eliminated the proton-activated currents while leaving normal responses to CAPS and low pH potentiation. Its mutation exhibited a weaker response to heat in amplitude, however, the thermal activation threshold was unchanged.	[192]
C634	G, S, W	It is involved in DTT interaction, its mutation and when associated with C616G and C621G significantly reduced DDT potentiation without having any effect on the CAPS, heat or voltage gating of the channel. Its mutation also enhanced the sensitivity to the acylpolyamine toxins AG489 and AG505, and caused toxicity when expressed in *Saccharomyces cerevisiae.*	[78,112,177,200,207]
E636	Q, W	The residue is a specific contributor to the CAPS response without affecting the proton or thermal sensitivity. Its mutation dramatically decreased the sensitivity to the acylpolyamine toxins AG489 and AG505.	[43,165,200]
F638	W	Its mutation enhanced the sensitivity to the acylpolyamine toxins AG489 and AG505.	[200]
F640	A, C, D, E, G, H, I, K, L, M, N, P, Q, R, S, T, V, W, Y,	The residue is critical for heat activation. Its mutation was constitutively active, and caused strong toxicity when expressed in *Saccharomyces cerevisiae.* Its mutation also enhanced the sensitivity to heat and CAPS, and abolished the proton potentiation of the channel. This mutation affected gating rather than permeation properties of the channel.	[177]
T641	S	The residue is involved in acid activation and potentiation. Its mutant displayed large constitutive channel activation and caused toxicity when expressed in *Saccharomyces cerevisiae.*	[177]
D646	N, W	The residue is a specific contributor to the CAPS response without affecting the proton or thermal sensitivity, and it is engaged in inhibition by RuRed. Its mutation reduced the permeability of divalent cations and dramatically decreased the sensitivity to the acylpolyamine toxins AG489 and AG505.	[43,165,176,197,213]
L647	W	Its mutation enhanced the sensitivity to the acylpolyamine toxins AG489 and AG505.	[200]
E648	A, Q	The residue is a specific contributor to the CAPS response without affecting the proton or thermal sensitivity. Its mutation reduced the proton-activated currents significantly, without altering the heat- or CAPS-evoked responses, and without eliminating the ability of protons to potentiate the responses to these stimuli.	[43,165]
F649	A, W	Its mutation caused reduced DkTx responses, and enhanced the sensitivity to the acylpolyamine toxins AG489 and AG505.	[198,200]
T650	S	The residue is involved in proton-induced potentiation. Its mutation caused large constitutive channel activation with abolished pH sensitivity. Its mutation caused toxicity when expressed in *Saccharomyces cerevisiae.*	[177]
E651	Q, W	The residue is important in pH activation. Its mutation dramatically decreased the sensitivity to the acylpolyamine toxins AG489 and AG505.	[43,192,200,213]
N652	D, T	The residue plays essential roles in the heat response without affecting the CAPS responses or the desensitization of the channel. Its mutation reduced the heat responses in amplitude and shifted them to higher temperatures. Its mutation also caused gain of function and strong toxicity when expressed in *Saccharomyces cerevisiae.*	[167,177]
Y653	T	The residue plays essential roles in the heat response without affecting the CAPS responses or the desensitization of the channel. Its mutation reduced the heat responses in amplitude and shifted them to higher temperatures.	[167]
K656	E, Q	Its mutation caused toxicity when expressed in *Saccharomyces cerevisiae.*	[177]
A657	P, W	The residue is critical for DkTx binding, its mutation showed loss of DkTx sensitivity.	[197]
V658	A	The residue is involved in acid potentiation. Its mutation showed a potentiation effect under moderately acidic conditions. Its mutation also caused toxicity when expressed in *Saccharomyces cerevisiae.*	[177]
F659	A, C, E, H, I, K, L, S, T, V, W, Y	The residue is involved in proton activation, and is a key integrator of voltage sensing, proton activation and potentiation. Its mutation caused lack of both voltage-dependent proton activation and potentiation, whereas activation by heat or CAPS was preserved. Its mutation caused reduced DkTx responses and caused toxicity when expressed in *Saccharomyces cerevisiae.*	[177,194,195,198,199]
Y666	A	Its mutation resulted in non-functional channel.	[175]
I668	A	Its mutation reduced CAPS sensitivity, heat-induced current responses and heat-potentiated CAPS currents.	[175]
L669	A	The residue is involved in heat activation but not in CAPS activation, nor in the heat potentiation of the CAPS currents.	[175]
Y671	A	The residue might contribute to allosteric coupling between temperature- and CAPS- dependent activation mechanisms. Its mutation affected the heat-induced current responses, lowered the CAPS EC_50_ value of the channel. Responses to CAPS were not potentiated by heat in the mutant containing channel, in fact CAPS-evoked responses were inhibited by heat. The residue is involved in the regulation of permeability of divalent cations, it gates the access of smaller cations.	[169,175,176,178,197]
I672	A	Its mutation reduced CAPS sensitivity, heat-induced current responses and heat-potentiated CAPS currents.	[175]
L673	A, I	Its mutation caused gain of function and strong toxicity when expressed in *Saccharomyces cerevisiae.* Its mutation also reduced CAPS sensitivity, heat-induced current responses and heat-potentiated CAPS currents.	[175,177]
L674	A	Its mutation reduced CAPS sensitivity, heat-induced current responses and heat-potentiated CAPS currents.	[175]
L675	A	Its mutation reduced CAPS sensitivity and heat-potentiated CAPS currents.	[175]
N676	A, F	Its mutation influenced the ability of CAPS and RTX to activate TRPV1 without changing the response of the channel to protons. Its mutation to Ala resulted in non-functional channel.	[166,175]
M677	A	Its mutation in a triple mutant (N676F/M677A/L678P) influenced the ability of CAPS and RTX to activate TRPV1 without changing the response of the channel to protons. Its mutation alone blunted heat-induced current responses without a significant change of CAPS- or heat-potentiated CAPS currents.	[166,175]
L678	A, P	Its mutation influenced the ability of CAPS and RTX to activate TRPV1 without changing the response of the channel to protons. Its mutation also blunted heat-induced current responses, with a leftward shift in the temperature threshold.	[166,175]
I679	A	Its mutation retained normal sensitivities to CAPS and heat, although it completely removed their mutual potentiation.	[175]
L681	A	The residue is involved in the regulation of permeability of divalent cations, it regulates the permeability of large cations.	[169,175,178]
M682	A	Its mutation caused impaired CAPS- and heat- activation, and significantly reduced their mutual potentiation.	[175]
E684	G, V	Its mutation caused gain of function and strong toxicity when expressed in *Saccharomyces cerevisiae.* Its mutation caused significant response to pH 6.4 (threshold concentration for proton-activation).	[177]
I689	V	Its mutation caused gain of function mutation and weaker toxicity when expressed in *Saccharomyces cerevisiae.*	[177]
K694	A	The residue participates in PIP2 binding.	[206]
K698	A	The residue participates in PIP2 binding.	[206]
Q700	A	The residue participates in PIP2 binding.	[206]
R701	A	The residue participates in PIP2 binding.	[191,206]
T704	I	The residue is a CaMKII phosphorylation site. PMA decreases the binding of [^3^H]RTX to TRPV1 through interaction with this residue. When associated with S502A, its mutation lost the ability to be activated by CAPS and lost the ability of vanilloid binding.	[46,85,91,115,174]
K710	A, R	The residue participates in PIP2 binding. Its mutation caused gain of function and strong toxicity when expressed in *Saccharomyces cerevisiae.*	[177,191,206]
Q727		The residue has key roles in heat activation.	[191]
F742	S	Its mutation caused gain of function and strong toxicity when expressed in *Saccharomyces cerevisiae.*	[177]
W752		The residue has key roles in heat activation.	[191]
E761	K, Q, Δ	Its deletion or mutation blocks RTX binding and proton- and CAPS-induced currents without affecting TRPV1 activation by heat.	[16,175,176]
K770	A	The residue participates in PIP2 binding.	[206]
R771	A	The residue participates in PIP2 binding.	[206]
R778	A	The residue has key roles in PIP2 binding.	[201,206]
R781	A	The residue has key roles in PIP2 binding.	[201,206]
R785	A	The residue participates in PIP2 binding.	[201,206]
W787	R	Its mutation caused gain of function and strong toxicity when expressed in *Saccharomyces cerevisiae.*	[177]
L792	P	Its mutation caused gain of function mutation and weaker toxicity when expressed in *Saccharomyces cerevisiae.* Its mutation caused significant response to pH 6.4 (threshold concentration for proton-activation).	[177]
L796	P, V	Its mutation caused gain of function and strong/weaker toxicity when expressed in *Saccharomyces cerevisiae.*	[177]
S800	A	Its phosphorylation by PKC potentiates CAPS, acid, and thermal responses.	[78,85,209]

### Mutations affecting agonist action and/or binding

#### CAPS and RTX

Jordt et al. provided molecular evidence that the mutation of **E600** caused enhanced sensitivity to CAPS [43]. In the experiments by Jordt et al., replacement of Glu by Gln (**E600Q**) produced a greater than 10-fold leftward shift in the CAPS dose–response curve, with EC_50_ values of 520 and 40 nM for wild type and mutant receptors, respectively (Figure 1). Moreover, channels bearing a Lys at this position (**E600K**) exhibited even higher agonist sensitivity, showing saturated current responses at 50 nM CAPS. Introduction of neutral or positive residues at the **E600** site potentiates responses to CAPS, whereas introduction of a residue with lower pKa (**E600D**) decreases the channel sensitivity to this stimulus [43]. Welch et al. identified **E636**, **D646** and **E648** as specific contributors to the CAPS response without affecting the proton or thermal sensitivity. E636Q, D646N and E648Q had a threefold greater sensitivity to CAPS than the wild type TRPV1 channels [165].

A triple mutant (**N676F/M677A/L678P**) was reported by Kuzhikandathil et al. to disrupt the ability of CAPS and RTX to activate TRPV1, while retaining the ability to respond to protons [166], suggesting that distinct amino acids that are close to or in TM6 control the gating in response to several modes of TRPV1 activation.

Deletion of **R114** and **E761** in the N- and C-termini can block CAPS-induced currents without affecting TRPV1 activation by heat [16].

**F489Y** resulted in a rightward shift of the CAPS concentration of half-maximal activation (EC_50_) [167]. Jordt et al. [168] conducted a search for residues involved in the action of CAPS on the basis of the differences between avian TRPV1 and mammalian (rat and human) orthologues [169], and found eight amino acids in the vicinity of TM3 that differ between the two forms, which may account for the species-specific sensitivity to CAPS (**F507, Q519, F522, M523, S532, K535, E536** and **T550** residues of rTRPV1 corresponding to **I515, N527, L530, L531, C540, E543, L544** and **A558** of chicken TRPV1, respectively). Exchange of any one of the **Q519, F522, M523, S532, K535** or **E536** residues for the chicken counterpart N, L, L, C, E or L, respectively, is sufficinet to ablate the vanilloid sensitivity of rTRPV1. They investigated the TM2-4 regions for sites involved in CAPS, RTX or proton action and observed that substitutions at two positions (**R491G** in TM2 and **S512F** in TM3) led to dramatic reductions in both proton- and CAPS-evoked currents. However, the introduction of other mutations at these positions (**S512A, S512T** or **R491E**) resulted in a greater preservation of functionality, and in each case a greater reduction in CAPS sensitivity was observed compared to extracellular protons. The mutant **S512Y** displayed little response to CAPS up to 100 μM, and no detectable specific binding of RTX. Nevertheless, the proton sensitivity was retained, as were the responses to noxious heat, albeit with a slightly higher thermal threshold (>48°C) and smaller maximal amplitudes compared to wild type receptors. An extended mutational analysis of the conserved residues adjacent to **S512** (**D509**, **S510**, **Y511**, **E513** and **I514**) revealed the most significant effects with the mutant **Y511A**. Despite lacking any significant CAPS sensitivity, this channel exhibited normal heat- and proton-evoked responses, with a thermal threshold and current amplitudes that were indistinguishable from those of the wild type receptor. In tests of whether the aromatic nature of the residue at position 511 is essential for ligand binding, substitution with a Phe (**Y511F**) was found to have only moderate effects, whereas substitution with a non-aromatic Cys (**Y511C**) again eliminated the CAPS sensitivity [168].

Jung et al. [16] identified two regions near **Arg114** and **Glu761** in the cytosolic tails of TRPV1 that determine ligand binding. Because the **Arg114** and **Glu761** residues are charged, it is likely that these charges are necessary for vanilloid binding. When positively charged **Arg114** was replaced by a neutral amino acid, Ala, the mutant (**R114A**) elicited a CAPS-induced current comparable with that of the wild type **TRPV1**. However, when the Arg at 114 was replaced by negatively charged glutamate (**R114E**), a significant reduction in CAPS-induced current was observed with no apparent specific [^3^H]RTX binding. Because the adjacent amino acid, **R115** is also positively charged, it was also replaced with Asp (**R115D**). The **R115D** mutant also abolished the CAPS-sensitive currents, indicating that charge at Arg-115 contributes equally to activation by CAPS. When the negatively charged Glu at 761 was changed to Gln, a neutral amino acid retaining a similar structure, the mutant (**E761Q**) elicited a great reduction in I_cap_ (~98.0% reduction), and had no specific binding for [^3^H]RTX. Furthermore, when the Glu at 761 was substituted with positively charged Lys, the mutant (**E761K**) showed no current response to CAPS or ability to bind [^3^H]RTX. **R114E** and **E761K** elicited current responses to heat, but not to acid (pH 5.5).

These results reflect the necessity of the negative charge of Glu at **761** for ligand recognition and the fact that the positive charge at **R114** determines the ligand binding to the channel, but to a lesser extent than the charge at **E761**[16].

Chou et al. [170] showed the residue in position **547** to be a key contributor to RTX binding of TRPV1 by exchanging a single amino acid between the human and rat counterpart. Systematic replacement of the amino acids in the TM1 to TM4 domain of hTRPV1 with the corresponding rTRPV1 residues identified a single conservative substitution of Met for Leu (**M547L**) at position 547 that accounted for the species difference in RTX binding. The amino acid at this position also affected the potency of the antagonists I-RTX and capsazepine for inhibiting [^3^H]RTX binding and the agonist response to RTX. The **M547A** mutation in rTRPV1 reduced RTX affinity to the same degree as M547L (≈30-fold). A more radical mutation, **M547Q**, decreased potency to a smaller degree than either **M547A** or **M547L**. In the case of **L547A** and **L547Q** mutations in the hTRPV1 no [^3^H]RTX binding and little channel activity were detected even though protein was seen on western blots. The human **L547M** mutation has a higher affinity for RTX than wild type rTRPV1, and similarly the rat **M547L** displayed a lower affinity than wild type hTRPV1 [170]. **M547**, **W549** and **T550** in the S4 segment participate in ligand interactions [171,172]. **F489** proved to take part in the CAPS activation of the channel [167], probably because of its close proximity to a domain containing mutations **R491**, **Y511**, and **S512** shown to be implicated in CAPS sensitivity [168,173].

When associated with **T704I**, **S502A** was found to lose the ability to be activated by CAPS and lose the ability of vanilloid binding [85,91,174].

Rabbit oTRPV1 can be activated by heat (45°C) and protons (pH 5), but it is 100-fold less sensitive to vanilloid activation than hTRPV1 or rTRPV1, and oTRPV1-transfected HEK293 cells did not exhibit any specific [^3^H]RTX binding. Gavva et al. [171] constructed a rat-rabbit chimera of TRPV1 by the transfer of TM3 through 4 (**S505-T550**) from rTRPV1 to oTRPV1. The chimera displayed enhanced sensitivity to vanilloids, similarly to rTRPV1. Additionally, a human-rabbit chimera was created by transferring the **S505-T550** from hTRPV1 to oTRPV1. The functional analysis again showed that this chimera also gained sensitivity to CAPS, further confirming that the TM3-4 region is responsible for vanilloid sensitivity.

On the basis of the 10 differences found between rTRPV1 and oTRPV1 and the six between hTRPV1 and oTRPV1, Gavva et al. mutated the residues that in rabbit differ from those in both rTRPV1 and hTRPV1 (**A505S**, **A520S**, **C534R**, **T540S** and **I550T**). Changing the single residue at 550 in rabbit to the corresponding residue found in rTRPV1 and hTRPV1 (**I550T**) was sufficient to confer a gain of function for activation by CAPS.

Changing the residues **A505S**, **A520S**, **C534R** and **T540S** individually or in various combinations did not cause any changes in the responses of oTRPV1 to vanilloids.

For a better understanding of the biophysical requirements at position **550**, Gavva et al. explored several polar and hydrophobic substitutions. A gain in CAPS sensitivity was observed when Ser was introduced instead of Thr (**I550S**), the small non-polar Ala (**I550A**) resulted in only a partial gain of CAPS sensitivity, while thiol group containing-residue Cys (**I550C**) resulted in a very small gain in oTRPV1 CAPS sensitivity. Introduction of Tyr (**I550Y**) with its bulky phenolic side-chain at this position resulted in a complete loss of TRPV1 response to vanilloid, proton or heat activation, although the expression level of this mutant remained comparable to the others. To verify further that the **T550** found in native rTRPV1 hTRPV1 contributes to the vanilloid sensitivity of TRPV1, the effects of substitution of the natural Thr by the oTRPV1 Ile **550** (**T550Y**) residue were examined. ^45^Ca^2+^-uptake experiments revealed a 10-fold loss in sensitivity to CAPS of rTRPV1-**T550I**, and a 40-fold loss in sensitivity of hTRPV1-**T550I**.

Gavva et al. [171] confirmed the finding of Jordt and Julius that **T511** is critical for vanilloid sensitivity. Both rTRPV1-**Y511A** and hTRPV1-**Y511A** had lower vanilloid sensitivity. They tested the CAPS sensitivity of the oTRPV1 double mutant containing **I550T** (gain of function) and **Y511A** (loss of function), i.e., oTRPV1-**Y511A**/**I550T**. Compared with oTRPV1-**I550T**, the reduction in CAPS sensitivity of oTRPV1-**Y511A**/**I550T** in the ^45^Ca^2+^ uptake assay was >100-fold. In fact, the magnitude of the loss in CAPS sensitivity by **Y511A** was greater than the gain seen in **I550T**. The **T550I** mutation resulted in the CAPS dose-response curve shifting 10-fold to the right, relative to the control, without reducing RTX sensitivity in the ^45^Ca^2+^ uptake assay. However, [^3^H]RTX specific binding was significantly reduced in rTRPV1-**T550I**-transfected cells. A series of single point mutations were introduced into oTRPV1 (**M514I, A525V, T526S, H533Q** and **L547M**) to mimic the residues in rTRPV1, which has been shown to display the highest RTX-binding affinity. Replacement of the oTRPV1 residues at **M514I**, **A525V**, **T526S** and **H533Q** individually did not alter the oTRPV1 response to CAPS or RTX. The single residue change **L547M** in oTRPV1 resulted in a selective gain of ~30-fold higher sensitivity to RTX with no apparent change in CAPS sensitivity in ^45^Ca^2+^-uptake assays. oTRPV1-**L547M** demonstrated greater sensitivity to RTX, but failed to show any measurable [^3^H]RTX binding, and it was therefore hypothesized that **L547M** contributes to RTX sensitivity, but requires additional residues such as **T550** to attain the affinity needed for measurable [^3^H]RTX binding above the assay background. A reverse mutation in rTRPV1 (**M547L)** displayed similar responses to CAPS and RTX in the functional ^45^Ca^2+^-uptake assay. To test whether oTRPV1 might require both **T550** and **M547** to attain measurable [^3^H]RTX binding, the oTRPV1 **L547M**/**I550T** double mutant was constructed and its vanilloid sensitivity was examined in functional ^45^Ca^2+^-uptake and [^3^H]RTX binding assays. As predicted, oTRPV1-**L547M**/**I550T** exhibited strong [^3^H]RTX binding together with only a slight increase in functional sensitivity to CAPS and a somewhat greater increase for RTX. This demonstrates that **Met547** and **Thr550**, as present in native rTRPV1, are required for measurable [^3^H]RTX binding in oTRPV1. oTRPV1-**I550T** and **L547M**/**I550T** resulted in slight changes in sensitivity to pH 5.5. Gavva et al. hypothesized that **Y511**, **M547** and **T550** may be present in the binding pocket and are important molecular determinants for vanilloid sensitivity [171].

Johnson et al. [173] investigated the role of two residues (**S512** and **M547**) identified earlier to be responsible for major species-specific differences in vanilloid activity in the response of the channel to a variety of ligands. Their initial studies confirmed the clear species-specific differences in RTX potency described previously by Chou et al. [170]. RTX activated wild type rTRPV1 with a fourfold higher potency than the wild type hTRPV1. Johnson et al. switched the residues at position **547** between the two receptor homologs creating the **M547L** rat mutant and the **L547M** human one. **L547M** produced a significantly more RTX-sensitive channel with the EC_50_ value comparable with that of the wild type rat receptor. Conversely, when the reverse mutation was made in the rat construct (**M547L**) a loss of function was observed, although, the change was not significant.

The species-specific differences in CAPS sensitivity were also investigated. CAPS was found to be significantly more potent at the wild type hTRPV1 than at the wild type rTRPV1 channel. When the **547** residue was switched between the rat and the human constructs, the sensitivity to the agonist was again altered. In this instance, this mutation caused a significant decrease in potency in the human mutant (**L547M**) compared with the human wild type, with an EC_50_ value comparable with that of the wild type rTRPV1. As for RTX, when the reverse mutation was made in the rTRPV1 construct, there was no significant difference [173].

Susankova et al. [175] Ala-scanned the residues **Y666**-**G683** containing the most conserved region of the TRP protein family.

Three mutant channels (**Y666A**, **I672A**, **N676A**) showed no CAPS-evoked activity at all. These mutants were also pH insensitive, they were not activated in the absence of extracellular Ca^2+^, and they gave very small and nonspecific heat-induced membrane currents. **Y666A** and **N676A** possessing no any potentiated currents in response to 47°C and 30 μM CAPS applied together, proved to be non-functional mutants, suggesting that these two residues within the inner-pore region of rTRPV1 are critical for channel functionality.

Co-application CAPS (30 μM) and heat stimuli (43°C) caused efficient potentiation in mutants **I672A**, **L673A**, **L674A**, **L678A**, and **M682A** which were functionally silent or only barely responded to CAPS at base temperature. Compared with wild type TRPV1 however, the current densities of the CAPS-induced responses measured at 47°C were significantly reduced in: **I668A**, **I672A**, **L673A**, **L674A**, **L675A**, and **M682A**. These mutations but **L675**, whose heat-activation was unaffected, caused also significantly impaired heat- and CAPS-activation.

**Y671A** dramatically lowered the CAPS EC_50_ value in the experiments of Mohapatra et al. [176].

Susankova et al. [175] further studied this mutant and reported that responses to CAPS were not potentiated by heat, but in contrast to the other mutants and the wild type channel, CAPS-evoked responses were consistently inhibited by heating above 30°C. Susankova et al. reasoned that the loss of potentiation in Y671A might be attributable to changes in agonist sensitivity. When the channels were desensitized by repeated applications of CAPS (1μM and/or 30μM), the heat-induced potentiation reappeared. Y761 might contribute to allosteric coupling between temperature- and CAPS- dependent activation mechanisms.

Susankova et al. evaluated the changes in CAPS responsiveness evoked by Ala mutations by comparing their relative sensitivities at 1 and 30 μM CAPS at 47°C.

An alternating pattern was found with unaltered sensitivity to CAPS in **T670A**, **L675A**, **G683A**, and wild type channels and a reduced but not abolished CAPS sensitivity in mutations **I668A**, **Y671A**, **I672A**, **L673A**, **L678A**, and **M682A**. Interestingly, the mutation of Leu **673** to Ile (**L673I**) in the experiments of Myers et al. [177] however caused a channel with elevated basal activity and significant cytotoxicity.

To further assess the maximal CAPS-induced responses in the mutants, Susankova et al. compared the inward currents measured at 47°C in the absence and presence of a high concentration of CAPS. Except for **Y666A**, **N676A**, and **I679A**, the inward currents were significantly greater in the presence of 30 μM CAPS than in its absence. The lack of mutual potentiation between the two stimuli in the former two mutants was caused by their insensitivity to CAPS. Notably, the mutation **I679A** retained normal sensitivities to both stimuli, although it completely removed their mutual potentiation at 47°C. In several residues (**V667A**, **T670A**, **I672A**, **L673A** and **L675A**), the degree of the CAPS-induced increase in the amplitude of the inward current at 47°C was clearly reduced, although these changes did not achieve statistical significance.

Changes induced by individual mutations in the 1 μM versus 30 μM CAPS-induced current relationships measured at 47°C might reflect the changes either in CAPS sensitivity or in the allosteric linkage between CAPS and heat sensor movements and channel opening. Specifically, mutation **I679A** appeared to cause a decrease in the synergistic interaction of CAPS and heat with the TRPV1 channel.

Susankova et al. denoted that the periodicity observed in the relationships between the maximal activation capacities obtained for 1 and 30 μM CAPS at 47°C supports the hypothesis that there exists a structural explanation for the gating of the TRPV1 channel by chemical stimuli. The pattern of sensitivity to CAPS (every three to four substitutions) is consistent with an α-helical structure contributing to CAPS-induced channel gating. Similar pattern of residues involved in the CAPS sensitivity can be observed in TM3 and TM3-TM4 linker region identified by Jordt and Julius [168]. The repetitive patterns of CAPS sensitive residues apparent in both papers however seem to fit better for a 3.4 residues per turn of the helix than for a 3.6 one. This may further support the finding of Salazar et al. [178] who reported that the TM6 of TRPV1 represent amphipathic helix with 3.4 residues per turn and a P(φ) value of 107° rather than α-helix with 3.6 residues per turn and a P(φ) value of 100°. Considering the above mentioned findings, all the TM helices of TRPV1 might be regarded to have the same structure [175].

Boukalova et al. [179] found that the **E570Q** mutation accelerated the rate of activation of the channel. In contrast, a significantly lower rate of activation was observed in mutated rTRPV1 channels containing mutations **R557A**, **M581T**, **D576R**, **Q560H**, **R557K** and **E570R**, indicating contribution of the transduction of the CAPS-binding signal to the opening of the pore. The estimated deactivation time (the time taken for the current to decrease to 50% of its level before removal of the CAPS) was markedly longer in **R557K** as compared with the wild type, but not in **R557A** or **R557L**, indicating that the specific side-chain properties of **R557**, and not only a positive charge at this residue, are important for the deactivation gating process. In **R557A**, **R557K**, **Q560H** and **M581T**, the second response to 1 μM CAPS reapplied after an apparent wash-out had a faster onset than in the wild type, suggesting an incomplete deactivation process. The time courses of the CAPS-induced whole-cell currents through **R557A**, **R557L**, **E570R**, **D576R**, **R579A** and **R576R**/**R579D** closely resembled those of wild type TRPV1. In contrast, **R557E**/**E570R** exhibited slower activation and deactivation kinetics. A significantly faster offset of CAPS-dependent responses was detected in **E570A** and **R576R**/**R579E**. The mutations causing similar defects in the voltage-dependence affected the chemical sensitivity of TRPV1 very differently [179].

Mutation studies by Lee et al. [180] and other groups, along with comparisons of TRPV1 variants from species sensitive or insensitive to vanilloids, have identified important residues for ligand binding, such as **Tyr511**, **Met547** and **Thr550**[168,170,171].

Lee et al. [180] generated a series of rTRPV1 mutants within the TM3 (**Y511A**, **Y511F**) and TM4 (**M547L**, **T550A**, **T550I**, **T550S**) regions, and performed docking studies with the prototypical agonists, CAPS and RTX, to assess their roles in ligand recognition. Their mutational studies based on the foregoing *in silico* docking experiments resulted in the discovery that the vanillyl moiety of CAPS oriented towards **Y511**, while the tail end extended towards **M547**. The vanillyl moiety formed π-π stacking and hydrophobic interactions with **Y511,** and H-bonding with **S512**. Moreover, the carbonyl group participated in H-bonding interactions with **Y511** and **L571**. Mutation of Tyr in position **511** to Phe (**Y511F**) had only a slight effect on the activity of CAPS, but its mutation to Ala (**Y511A**) caused loss of the π-π stacking and H-bonding capabilities, leading to a sharp decrease in CAPS activity. The mutation of **T550I** also evoked a significant decrease in CAPS activity, but the influence of the change of Thr to Ala (**T550A**) or Ser (**T550S**) was much weaker. This may reflect the bulky side-chain of Ile disturbing the binding of the nonenyl tail of CAPS. Although the hydrophobic nonenyl tail was oriented towards the upper hydrophobic region of the binding site, it did not fully occupy the hydrophobic region of the two shallow hydrophobic areas composed of **F543** and **M547** because it is linear and too short to reach both areas. These data indicate the relevance of the overall size, shape and/or hydrophobicity of the lipophilic side chain region for binding.

In the case of RTX, the vanillyl moiety appeared to form π-π stacking with **Y511,** as did that of CAPS. The importance of **Y511** in RTX binding was also confirmed by their mutation study. When Tyr **511** was mutated to Phe (**Y511F**), the binding affinity of RTX decreased less than fourfold, as the π-π stacking and hydrophobic interactions of the vanillyl group of RTX were maintained. As compared with the relatively short and linear tail of CAPS, the C13-propenyl group of RTX contributed to the hydrophobic interaction with **M547**, and its importance in RTX binding was also justified by the mutation studies. When Met was mutated to Ile (**M547I**), the binding affinity of RTX decreased more than 11-fold. This may be caused by the greater ability of **M547** than of Leu to extend to make the hydrophobic interaction with RTX. In addition, the C4-OH group of RTX seemed to fit well with the small side-chain of **T550** in addition to H-bonding with the residue. This docking result is in agreement with the mutation data that neither mutated **T550S** nor **T550A** caused any binding loss relative to the wild type, while **T550I** led to a drastic decrease (over 20-fold) in RTX-binding affinity. As with CAPS binding, the bulky side-chain of Ile could cause steric interference with the binding of RTX. It was noticeable that the orthophenyl group of RTX underwent hydrophobic interaction with **L515**. The ultrapotency of RTX might be due to the fact that it could occupy the binding site fully, taking maximum advantage of the multiple possible binding interactions with TRPV1. Since RTX has phenyl rings in both the 4-hydroxy-3-methoxyphenyl and lipophilic side chain regions and there are hydrophobic residues at both ends of the binding site, RTX could flip over and achieve a minor binding mode. In this case, the vanillyl moiety would point towards **M547** and participate in the hydrophobic interaction. Correspondingly, the orthophenyl group would orient towards **Y511**. The C20-ester seemed to take part in H-bonding interactions with **N551** and the C13-propenyl group formed the hydrophobic interaction with **L515**[180].

#### APB

Boukalova et al. [179] reported that the inward currents induced by 300 μM 2-APB were reduced in **R557L**, **R557K**, **Q560H**, **E570Q** and **E570R** and most strongly (>50-fold) in **K571E**, which was normal in all other aspects of TRPV1 activation (Figure 1).

#### Allicin

The TRPA1 channel, which is co-expressed in many of the same neurones as TRPV1, has been proposed to be the sole target for the actions of allicin [56,181,182]. However, other groups have reported that TRPV1 is also a target for the actions of this compound [57,183-185]. The agonist action of allicin was confirmed by Salazar et al. [186], who further demonstrated that the allicin behaves as a TRPV1 agonist through the covalent modification of a single Cys localized in the N-terminus of the channel at position C157 [187] (Figure 1).

### Mutations affecting heat activation and potentiation

The heat activation pathway is largely unknown, although the outer pore region, including the turret and the selectivity filter-to-S6 linker, is clearly part of the protein structure participating in the heat-induced conformational rearrangement. Intensive studies of thermo TRP channels have so far indicated numerous channel regions that contribute to temperature-dependent activation. Exchanging the intracellular C-termini between TRPV1 and the cold-activated TRPM8 channel was found to switch their sensitivity to heat [188]. This result is consistent with the earlier observation that deletion of the last 72 amino acids of the TRPV1 C-terminus influenced channel activation (though in a modality-independent manner) [84]. Furthermore, the intracellular segment between ANK and TM1 was recently proposed to serve as the thermal sensor for TRPV1 [189,190].

Introduction of neutral or positive residues at the **E600** site (**E600Q** and **E600K**) potentiated the responses to heat, whereas the introduction of a residue with lower pKa (**E600D**) decreased the channel sensitivity to it [43] (Figure 1).

Susankova et al. [175] performed Ala-scanning of the residues **Y666**-**G683**. The mutations **I668A**, **L669A**, **Y671A**, **I672A**, **L673A**, **L674A**, **M677A**, **L678A** and **M682A** blunted the heat-induced current responses. To obtain a more complete picture of how the individual mutations affect the heat sensitivity of the TRPV1 channel and to further characterize the specificity of their temperature-induced responses, the temperature threshold for activation and the temperature coefficient (*Q*_10_) from the Arrhenius plots of individual current–temperature relationships were quantified.

The most frequently observed phenotype was a leftward shift in the temperature threshold (**V667A**, **I668A**, **L669A**, **L673A**, **L674A**, **M677A**, **L678A** and **M682A**). Seven mutations (**T670A**, **Y671A**, **I672A**, **L675A**, **I679A**, **L681A** and **G683A**) had thermal thresholds no different from wild type TRPV1. Except for the **T670A**, **L681A**, and **G683A**, the mutations strongly reduced *Q*_10_ in all mutants tested.

Susankova et al. claimed that average temperature-dependent activation profile with the five peaks separated by four troughs at residues **I672**-**L674**, **N676**, **L678**, and **M682** might correspond an α-helical structure, which most likely represents the inner-pore region of the TRPV1 channel. The results of Susankova et al. also provide functional support for the role of the putative inner-pore region in controlling the gating of the vanilloid receptor TRPV1 channel.

**L669A** and **M677A** are significantly less sensitive to heat without a significant change of CAPS- or heat-potentiated CAPS currents suggesting that these residues are involved in heat activation of the channel, but not in potentiation by heat. **L678A** displayed a reduced sensitivity to heat and CAPS with an unaffected heat-potentiated current, suggesting a role of **L678** in the process of CAPS- and heat- activation, but not in the potentiation mechanism. This finding somewhat contradicts to the results of Kuzhikandathil et al. [166] who demonstrated **M677** to affect the ability of CAPS and RTX to activate TRPV1 without changing the channel's response to protons however working on a triple mutant (**N676F/M677A/L678P**) containing channel [175].

By generating a chimera between the TRPV1 and TRPM8 channels, in which the region **V686** to **W752** of TRPV1 was replaced by the same C-terminal region of TRPM8 (**V982** to **W1055**), Brauchi et al. [191] identified TRPV1 C-terminal amino acids **Q727** and **W752** as being the minimum portion able to turn TRPM8 into a heat receptor.

The mutations **N628K**, **N652T** and **Y653T** resulted in TRPV1 channels responding normally to CAPS and pH, but whose heat responses were reduced in amplitude and shifted to higher temperatures. Moreover, the time course of activation of these single point mutants was identical or very similar as compared with wild type TRPV1, suggesting that the desensitization was not strongly altered [167]. A double mutant **N652T/Y653T** and a triple mutant N628K/N652T/Y653T yielded receptors with CAPS, 2APB and pH EC_50_ values and maximal responses that were indistinguishable from that of wild type TRPV1, but with a further reduction in temperature responses [167]. The triple mutant (**N628K/N652T/Y653T**) exhibited altered heat-gating kinetics. Whilst the unitary conductance of the wild type TRPV1 and the triple mutant channel was identical, the triple mutant possessed channel openings of only short (<1 ms) durations, and the longer (~10 ms) ones proved to be completely absent. The triple mutant, however, is normally gated by CAPS [167].

The **T633A** mutant exhibited a weaker response to heat, reaching ~32% of the 1 μM CAPS current. In contrast with the change in peak activity, however, the thermal activation threshold coincided with that of the wild type (~42°C) [192]. A less severe perturbation to the turret region of the TRPV1 channel by deletion of its first 10 amino acids (^**Δ**^**G603-S612**) substantially and specifically affected the heat activation but not CAPS activation of the channel [190].

A significantly higher threshold for heat activation was detected by Boukalova et al. [179] for **E570A**. The **Y554F**, **Y555F**, **Q561H**, **E570Q**, **E570R**, **K571E**, **R575A** and **R579D** mutations left the threshold for heat activation unchanged.

### Mutations affecting acid activation and potentiation

Jordt et al. [43] demonstrated that **E600** serves as a fundamental regulatory site for the proton potentiation of vanilloid receptor activity over a physiologically relevant range (pH 6–8), but not in proton or heat activation. The **E600Q** mutants retained the ability to be activated by acidic conditions or heat, but differed from the wild type receptor in two significant ways (Figure 1). First, upon heat activation the initial heat stimulus produced a relatively large peak current response that was much closer to the final plateau value than that typically observed with wild type channels. Second, bath acidification failed to potentiate peak currents beyond this steady-state value. Similar results were obtained with oocytes expressing the **E600A** and **E600S** mutants. The pH dependence of thermal activation was related to the side-chain charge of the residue at position **600**: heat-evoked currents in the **E600D** mutant were potentiated only when the bath pH dropped below 6.5. The **E600H** mutants, on the other hand, showed continuous potentiation over the entire pH range tested (9.0 to 5.0), but the extent of potentiation was significantly less than that observed for wild type or **E600D** channels. For **E600Q** or other mutants having non-titratable amino acids at this position, the magnitude of the heat-evoked currents was largely independent of the extracellular pH. The **D601N** and **E648Q** mutants exhibited a phenotype characterized by reduced proton-evoked responses, with normal CAPS sensitivity. The **E648A** mutants demonstrated an even greater decrease in proton-activated current amplitudes, whereas the CAPS- or heat-evoked currents did not differ significantly from those of wild type or **E648Q** channels [43]. This finding is in direct contradiction to the one made by Welch et al., who identified E648 as specific contributor to the CAPS response without affecting the proton or thermal sensitivity [165]. **E458Q**, **D471N**, **E478Q**, **E536Q**, **H614Q**, **E636Q**, **D646N**, **E651Q** and **D654N** mutations were also tested by Jordt et al. [43], but no phenotype was reported in terms of acid activation or potentiation.) **E600V** caused the complete and specific loss of pH sensitivity [167].

Deletion of **R114** was found by Jung et al. [16] to abolish the sensitivity to acid. When the positively charged Arg at 114 was replaced by a neutral amino acid, Ala, the mutant (**R114A**) elicited an I_cap_ comparable with that of the wild type TRPV1. However, when the Arg at 114 was replaced by negatively charged Glu (**R114E**), a significant reduction (~97% reduction) in I_cap_ was observed with no apparent specific [^3^H]RTX binding.

Sutton et al. reported that the **S512Y** mutant caused a small but significant decrease in the ability of protons to gate the TRPV1 channel [193].

A mutation of hTRPV1-**L547M** by Johnson et al. [173] caused a decrease in the potency of protons, but no increase was seen when the reverse switch was made in the rat receptor (**M547L**).

**E651** was found to be important for pH activation [192]. Substitution of the residue **T633** by Ala abrogated low pH-activated currents, but the **T633A** mutant exhibited normal CAPS responses, including rapid activation kinetics and large steady-state currents. Furthermore, the potentiation by low pH was also retained, despite the loss of the low pH sensitivity for direct activation. Conserved residues on the N-terminal end of the pore helix were also mutated by Ryu et al., i.e., **Y627A** and **S629A**. Both mutants were functional and produced relatively normal responses to CAPS when applied either alone or in combination with mildly acidic pH. The mutants were also activated by low pH directly, albeit with a slightly smaller maximal current than their wild type counterparts. The data suggest that these residues may contribute to, but do not play a pivotal role in the proton activation of TRPV1 as **T633** does [192]. In the wild type counterpart, pH 5.5 evoked long bursts of activity, in which the openings were separated by brief closures. The **T633A** mutant instead showed rare spike-like openings. The mutation drastically slowed the opening rate at low pH. The significant shortening of the open time suggests that the mutation destabilizes the open conformation of the channel. **T633** was systematically mutated to others, including Y, R, Q, N, L, K, E, D, V, S and A, which span both polarity and size. Substitutions with polar residues such as Q, N and Y or the charged residues R, K, E and D all resulted in non-functional channels. The **T633S** mutation was functional, but with a significant reduction in low pH current and a slow activation by CAPS. However, the **T633V** mutation preserved the wild type responses in all aspects. On substitution with Leu, containing a larger hydrophobic side-chain, the channel became non-functional. Together, these results suggested that **T633** is involved in functional interactions in a compact hydrophobic environment. The size of the side-chain at this position is crucial. Replacing Thr by Ala, a smaller side-chain, was able to preserve the CAPS response while abrogating the low pH activation; a larger side-chain substitution at this position, on the other hand, became deleterious [192]. The **K535E**, **E536W**, **V538L** and **A539P** residues were clustered around the centre of the loop, and the mutations appeared to impact predominantly the proton responses. **V538L** resulted in no detectable current at pH 5.5, while retaining >93% of the wild type peak CAPS response. The CAPS response of **E536W** was also reduced; however, this mutation, as with **K535E** and **A539P**, involved substantial changes in the side-chain property. Such substitutions could produce non-local perturbations on the channel structure and consequently non-specific phenotype changes. A common feature of all these mutations is the significant, consistent reduction of the low pH current, suggesting that the region plays an important role in proton activation. **S532M**, **Q533E**, **S540L** and **M541L** exerted little effect on either CAPS or low pH responses. The CAPS activation and its potentiation by low pH were not altered by the **V538L**, **E536W**, **K535E** or **A539P** mutations at the macroscopic level. **V538L** displayed a marked (>90% suppression) reduction in the maximal activity elicited by pH 4.5. Also unlike **T633A**, which exhibited no measurable pH current over the entire pH range, **V538L** furnished a titration curve with a consistently increasing trend as the pH was lowered. It appears that the mutation weakened, but did not disrupt the pH gating completely. The **V538L** mutant also exhibited a robust heat response.

The function of the channel appeared to be quite sensitive to perturbations at position 538 (**V538L**, **V538A**, **V538G**, **V538I**, **V538T**). Even the relatively conservative substitution with Ala abrogated the low pH currents and also reduced the CAPS activity. Further reduction of the size of the side-chain with a substitution by Gly resulted in non-functional channels [192].

In the experiments by Myers et al. [177], wild type TRPV1 responded strongly to CAPS but displayed negligible current under basal conditions or in response to pH 6.4, which is at the threshold concentration required for proton-evoked activation at room temperature. In contrast, eight mutants (**H166R**, **I352N**, **I352T**, **Q560R**, **M562D**, **E684G**, **E684V**, **L792P**), displayed a significant response to pH 6.4, although no basal current was detected at pH 7.4 Mammalian cell patch-clamp experiments by Myers et al. demonstrated that F640L displayed a strong basal channel activity and substantial toxicity when expressed in HEK293 cells. Whereas the wild type channel was potentiated by exposure to pH 6.2, the **F640L** current was completely unaffected under these conditions. Addition of CAPS to **F640L**-expressing cells led to a marked increase in current, demonstrating that **F640L** channels are not maximally open in the basal state. Higher doses of protons could activate the mutant channel illustrating that while the mutant has lost the ability to be potentiated within a certain pH range, its proton activation has not been completely ablated. Weakly alkaline solution (pH 8.2) failed to reduce the **F640L**-mediated basal current. **N628D** and **V658A** showed a potentiation effect under moderately acidic conditions. Furthermore **T641S** and **T650S** mutants displayed large constitutive channel activation with relative insensitivity to pH 6.4 [177].

Wang et al. reported that the first four TMs (TM1-4) of TRPV1 dictate whether the activity of a fully CAPS-bound receptor can be further enhanced by protons, and a glutamate residue (**E536**) in the linker between TM3 and TM4 of hTRPV1 is critical in the modulation by protons and in the further stimulation of fully liganded TRPV1 [75,194].

Aneiros et al. [195] replaced amino acid **F660** in hTRPV1 (corresponding to **F659** in rTRPV1) with a variety of different amino acids (A, C, E, H, I, K, L, S, T, V, W, Y) to determine the side-chain contribution to the proton activation of TRPV1. Proton activation was ablated by all amino acid replacements with the exceptions of **F660Y** and **F660W**, the two alternative non-basic aromatic amino acids besides Phe. Replacing Phe with His (**F660H**), which contains a basic aromatic ring (imidazole), or non-aromatic amino acids caused complete loss of proton activation. However, **F660Y** demonstrated a reduced sensitivity to proton activation as compared with wild type TRPV1. Although less pronounced, the maximum effect values at 1 μM CAPS were also reduced relative to the wild type, while the CAPS EC_50_ values at pH 7.4 were comparable. Ca^2+^ flux and whole-cell patch-clamp experiments using HEK293 cells transiently expressing TRPV1 mutants or wild type TRPV1 demonstrated a complete lack of activation of the mutant **F660S** by protons. In contrast, **F660S** maintained responsiveness to CAPS. TRPV1 mutant **F660S** ablated proton activation, but not CAPS or heat activation. **F660A** neither significantly inhibited nor significantly potentiated CAPS responses in the presence of protons. **F660W** showed a reduction in sensitivity to proton activation as well as CAPS activation similarly to **F660Y**. These data suggest that a non-basic aromatic amino acid at position **660** is essential for proton activation. A non-aromatic amino acid or His at position **660** seems to be tolerated for the channel to be functional in the CAPS activation mode; a non-basic aromatic side-chain, however, appears to be required to maintain activation by protons. The loss of activation by protons when **F660** is replaced with a charged amino acid and the absence of a titration phenotype suggest that Phe is critical for the transduction of proton-mediated gating rather than voltage or proton sensing. Aneiros et al. concluded that the proton activation and potentiation of TRPV1 are both voltage-dependent and that amino acid **660** is the key residue regulating the proton-mediated gating of hTRPV1 [195].

### Mutations affecting voltage sensing

Susankova et al. [175] also checked the Ala-scaned region of **Y666**-**G683** for mutants disturbing the voltage sensor function of the channel. To compare the effects of mutations on voltage-dependent activation, Susankova et al. used a voltage step protocol from −140 to +140 mV (increment, +20 mV) at 25°C and normalized the resulting current–voltage relationships at +60 mV for each cell.

Except for the **T670A**, **Y671A** and **M677A**, the Ala mutations led to a rightward shift of the activation curve and a concomitant decrease in the equivalent charge *z*, reflecting a decrease in channel activity at more negative holding potentials (Figure 1).

These data indicate that most of the Ala mutations modulate the gating of the TRPV1 by shifting the voltage dependence toward more positive membrane potentials. Temperature- and voltage-dependent mechanisms underlying the TRPV1 channel activation do not need to be strictly coupled [175].

Voets et al. demonstrated the roles of **K571**, **R575** and **R579** in voltage sensing using charge-neutralizing mutations in TM4 and the TM4-TM5 linker of human TRPM8 [172,196].

In the experiments of Boukalova et al. [179], the half-maximal activation voltage (V_1/2_) of wild type TRPV1 was 154 ± 4 mV, and V_1/2_ was shifted towards less depolarizing voltages for **R557K** (97.1 ± 4 mV), **G563S** (78 ± 2 mV), and **M581T** (122 ± 11 mV). Mutations **Y554A**, **Y555S**, **E570L**, **R557E** and **R579E** led to a complete loss of function, but three charge-swapping double mutants (**R557E**/**E570R**, **D576R**/**R579E** and **D576R**/**R579D**) exhibited measurable voltage-dependent activity, indicating a partial recovery of the functionality of the **R557E** and **R579E** mutant channels. To reveal the voltage-independent component of TRPV1 gating, Boukalova et al. [179] quantified V_1/2_ and the percentage of the voltage-independent component of gating from currents obtained in the presence of 10 μM CAPS. Significant changes in the apparent gating valence were detected in **E570R** and **D576R**/**R579D**, indicating that the S4-S5 linker may increase its contribution to voltage sensing when CAPS is present. **R557A**, **R557L**, **D576N** and **D576R**, which were only weakly voltage-dependent under control conditions, became as voltage-sensitive as wild type TRPV1 in the presence of CAPS, suggesting a preserved or even increased allosteric effect between these two stimuli. Relative to wild type TRPV1, the percentage of the voltage-independent component of CAPS-induced gating was found to be strongly reduced in **R557A**, **R557L**, **E570A**, **R579A**, **R579D**, **R557E**/**E570R** and **D576R**/**R579E**. In contrast, this component was predominant in **E570Q** and **E570R**. **G563S** possesses an enhanced voltage-dependent activity; inward currents induced by 1 μM CAPS exhibited slow activation kinetics and an incomplete deactivation that was fully blocked by 1 μM RuRed. **G563S** was not responsive to a temperature ramp (from 25°C to 48°C) and was only weakly sensitive to 300 μM 2-APB. These data suggest that the mutation **G563S** stabilizes the open conformation of the channel and thus **G563** in TRPV1 could play an analogous role in channel gating as in TRPV3 [179].

The **F660S** mutant in hTRPV1 (corresponding to **F659S** in rTRPV1) was shown by Aneiros et al. to lack both voltage-dependent proton activation and potentiation, whereas activation by heat or CAPS was preserved [194,195].

### Mutations affecting antagonist or channel blocker action/binding

#### I-RTX

**S512** and **M547** were tested by Johnson et al. [173] for their potential involvement in I-RTX action.

I-RTX shows species-specific activity on TRPV1, as well. The effect of the **547** residue on the ability of I-RTX to antagonize the response of TRPV1 to 500 nM CAPS was also challenged. As for RTX, the antagonist I-RTX was found to be significantly more potent at the rat receptor versus human receptor. After substitution of the rat-specific Met into the human construct (**L547M**), I-RTX gained functional potency, although the converse change (**M547L**) showed little effect (Figure 1). Introduction of the Met residue thus enables the human receptor to interact more effectively with both agonist and antagonist alike in a manner that is not matched by Leu.

**S512Y** was found to convert I-RTX from an antagonist to an agonist with nanomolar potency, albeit with much lower efficacy than its counterpart (RTX) for the wild type channel. Other agonists such as CAPS or acidification with pH 5.8 was shown to enhance the agonist potency of I-RTX, producing a 20-fold decrease in the EC_50_ value [173].

#### RuRed

D646N was reported by Garcia-Martinez et al. to decrease the efficacy of RuRed to block the channel by 10-fold [197] (Figure 1).

### Mutations affecting spider venoms action/binding

Venoms from spiders, snakes, scorpions and cone snails can cause burning pain. Small peptides, named vanillotoxins (VaTx) and “double-knot” toxin (DkTx) were indentified as TRPV1 agonists from the venoms of the spiders *Psalmopeous cambridgei* and *Ornithoctonus huwena*, respectively. Alanin scanning of the region **S592**-**A665** revealed three sites (**I599A**, **F649A**, and **F659A**) where alanin substitutions caused reduced toxin responses (Figure 1). **A657P** and **A657W** also showed loss of toxin sensitivity. *Xenopus laevis* xTRPV1 also contains Pro at the extracellular boundary of the S6 domain, corresponding to amino acid **A657** of rTRPV1, and is insensitive to VaTx or DkTx, but responds to CAPS and low pH. The reciprocal mutant (**P663A**) conferred toxin responsiveness of the frog TRPV1. The quadruple mutant (**I599A/F649A/A657P/F659A**) completely eliminated toxin sensitivity [198,199].

The venom from the spider *Agelenopsis aperta*, a North American funnel web spider, contains potent inhibitors of TRPV1. Two acylpolyamine toxins, AG489 and AG505, inhibit TRPV1 from the extracellular side of the membrane [200]. To identify mutations that alter these toxin affinity, Kitaguchi and Swartz [200] Trp scanned the TM5-TM6 linker region from Y627 to E651, mutating 25 consecutive residues to Trp. Of these mutants, 15 resulted in channel expression that could readily be studied by using electrophysiological techniques, whereas 10 produced little or no CAPS-activated current and were not studied further. One residue in this region (D646) was also mutated to Asn. The D646W mutant did not yield functional channel. N628W, E636W, D646N and E651W dramatically decreased toxin affinity. Mutations of Y627W , C634W and to a lesser extent F638W, L647W and F649W enhanced the sensitivity to the toxin [200]. According to these results and previous studies on polyamine inhibitors of cation channels, AG489 seemed to be a pore blocker.

### Sites of action of negative or positive modulators

A number of studies have demonstrated that the cytoplasmic regions of TRP channels bind agonists and regulatory molecules such as ATP, CaM and PIP2 [14,95,107,201-204].

#### ATP

Kwak et al. [205] found that **D178N** substitution abolished the ATP-mediated upregulation of TRPV1. Mutations generated by Lishko et al. [14], **K155A** and **K160A**, and the double mutation **Y199A**/**Q202A** impaired the TRPV1-ARD interaction with ATP. TRPV1 channels with mutations in the ATP-binding site (**K155A**, **K160A**, or **Y199A**/**Q202A**) showed little tachyphylaxis, even in the absence of ATP, while the two negative control mutants (**R181A** and **K265A**) had essentially wild type behaviour (Figure 1). The lack of tachyphylaxis shown by the TRPV1 mutants was not due to an impaired CAPS sensitivity; in fact, the mutant channels were slightly more sensitive to CAPS than the wild type channel [14,46].

#### Ca^2+^/CAM

Ca^2+^/CaM has been reported to bind to peptides from the N-terminal region of TRPV1, and that the residues **189**-**221** are crucial determinants for binding [107]. Grycova et al. [206] found that the CaM-binding site overlapped with the PIP2-binding site in the C-terminal distal region (**L777**-**S820**) and that PIP2 interacted with the proximal region (**I688**-**K718**) of the TRPV1 C-terminal (Figure 1).

Lishko et al. [14] found that the TRPV1-ARD mutants **K155A** and **K160A**, which no longer bind ATP, did not interact with CaM in size exclusion chromatography, emphasizing that the binding surface on TRPV1-ARD is at least partially shared by both ligands.

The TRPV1-ARD **Y199A**/**Q202A** mutant, where residues important for interactions with the adenine moiety of ATP were mutated, formed a weaker complex with CaM that eluted earlier than the complex with wild type TRPV1-ARD, still had a 1:1 stoichiometric ratio, suggesting that the different elution properties may be due to an altered conformation or binding constant, or higher-order (e.g., 2:2) complex [14].

#### PIP2

PIP2 has been shown to physically interact with a C-terminal fragment of TRPV1 [203]. In the experiments of Brauchi et al. [191] the mutation of the positively charged **R701** and **K710** to Ala strongly affected the PIP2-dependent activation, shifting the dose–response curves to the right along the concentration axis [191] (Figure 1).

Grycova et al. [206] showed that two different PIP2-binding sites on the C-terminus **L777-S820** and the N-terminus **F189**-**V221** overlapped with the CaM-binding sites, and the third PIP2-binding site **K688**-**K718** occupied the TRP domain on the C-terminus, a highly conserved sequence among the members of the TRP ion channel family. The presence of PIP2 was reported to prevent the interaction of the distal region of the C-terminus with CaM, which could play an important role in the regulation of TRPV1. To identify the residues important for the binding of PIP2 to TRPV1-C-terminus, a set of point mutations was generated by Grycova et al., involving the single substitutions **R778A** and **R781A**, the double substitutions **K770A**/**R785A**, **R771A**/**R781A** and **R771A**/**R778A** and the triple substitutions **K770A**/**R778A**/**R785A** and **K770A**/**R781A**/**R785A**. The most striking effect was the total loss of binding affinity observed for the single mutant **R778A**, the double mutant **R771A**/**R778A** and the triple mutant **K770A**/**R778A**/**R785A**. Moreover, the **K770A**/**R785A** and **R771A**/**R781A** mutations decreased the binding affinity. The **K694A**/**K698A**/**K710A** triple mutant seemed to completely lose its ability to bind PIP2. Ala substitutions of the additional candidate residues in the highly conserved QRA region **Q700A**/**R701A** significantly attenuated its binding affinity for PIP2. These data show that the TRPV1 C-terminal proximal region (**K688**-**K718**) binds PIP2 directly with a high affinity and suggest that basic residues play a crucial role in the binding.

The steady-state anisotropy measurements by Grycova et al. confirmed that the region denoted as a CaM interaction site **F189**-**V221** binds PIP2 with high affinity. On the basis of their molecular model of the PIP2 interacting with the TRPV1 C-terminal distal region, Grycova et al. suggested that the phosphate head groups of PIP2 form polar interactions with positively charged Arg residues **R778**, **R781**, **R785**. PIP2 thus occupies the CaM binding groove containing **R771**, **R778**, **R781**, **R785** described previously [201]. Residues **R778** and **R781** were found to have key role in the binding of PIP2. Further combinations of Ala substitutions revealed that the TRPV1-CT distal region participates in PIP2 binding through a cluster of basic residues: the double and triple substitutions of **R771A**/**R778A** and **K770A**/**R778A**/**R785A** avoided PIP2 binding totally, and the **K770A**/**R785A** and **R771A**/**R781A** mutations suppressed this interaction partially. Site-directed mutation of **R701** Arg reduced the binding affinity for PIP2. The triple substitution at positions **K694A**/**K698A**/**K710A** had the most pronounced effect, preventing PIP2 completely from binding to this region. The regions **F189**-**V221** within the N-terminus and **K688**-**K718** and **L777**-**S820** within the C-terminus are involved in PIP2 binding. Interestingly, the **F189**-**V221** and **L777**-**S820** regions overlap with the CaM-binding sites, suggesting that CaM and PIP2 compete for the same binding site, which might have implications for regulation of the channel function. **R778A** was found to have the key role in the interaction. This single mutation leads to a total loss of binding affinity of the distal C-terminal region [206].

#### Reducing agents

DTT, an agent that maintains the -SH groups of Cys in a reduced state, has been reported to facilitate membrane currents through TRPV1 when applied from the extracellular face of the channel, by interacting with the residues at positions **C616**, **C621** and **C634** in the loop between the fifth and sixth transmembranal domains [207] (Figure 1).

Site-directed mutagenesis experiments in the pore loop have identified **C621** as the residue responsible for the extracellular modulation of TRPV1 by reducing agents [112]. Mutations **C616G** and **C634G** did not affect DDT potentiation at 45°C, but **C621G** and the triple mutant **C616G/C621G/C634G** significantly reduced DDT potentiation without having any effect on the CAPS, heat or voltage gating of the channel [78,112].

#### Cholesterol

Using measurements of CAPS-activated currents in excised patches from TRPV1-expressing HEK293 cells, Picazo-Juárez et al. [208] showed that enrichment with cholesterol, but not its diastereoisomer epicholesterol, markedly decreased wild type rTRPV1 currents in the presence of CAPS, elevated temperature or voltage.

Substitutions in the S5 helix by Picazo-Juárez et al. [208], **R579D** and **F582Q**, decreased the cholesterol response and **L585I** was insensitive to cholesterol addition (Figure 1). Two hTRPV1 variants, with different amino acids at position **585**, displayed different responses to cholesterol, with hTRPV1-I585 being insensitive to this molecule. However, hTRPV1-**L585** was inhibited by cholesterol addition similarly to rTRPV1 with the same S5 sequence (containing **L585**). In the absence of CAPS, cholesterol enrichment also inhibited the TRPV1 currents induced by elevated temperature and voltage.

The amino acids in positions **K571**, **R575** and **R579** were confirmed to be involved in TRPV1-lipid interactions [172,191].

### Mutations of phosphorylation sites

Phosphorylation by PKC, which potentiates CAPS, acid, and thermal responses in TRPV1 channels, occurs at two target Ser residues (**S502** and **S800**) [78,85,209] (Figure 1). Residues located in the N-terminus of TRPV1 (**S116** and **T370**) are phosphorylated by PKA and have been implicated in desensitization [85,176,210] whereas residues **T144**, **T370** and **S502** have been implicated in the sensitization of heat-evoked TRPV1 responses when phosphorylated by PKA [78,83]. Phorbol 12-myristate 13-acetate (PMA), a PKC-activating phorbol, was observed to decrease the binding of [^3^H]RTX to TRPV1 [115] through interaction with **Y704** in the C-terminus [46,85].

The site-directed mutation of residue **S116A** performed by Wang et al. [90] was reported to block both the phosphorylation of rTRPV1 by PKCμ and the enhancement by PKCμ of the response of rTRPV1 to CAPS. **Ser116** is also a major phosphorylation site in TRPV1 for PKA, and this site has been shown to be involved in TRPV1 desensitization [116].

Numazaki et al. [174] observed that, when **S502** and **S800** residues were replaced with Ala, the TRPV1 activity induced by CAPS, protons or heat was eliminated [78]. **S502A** was found to significantly reduce PMA enhancement of CAPS-evoked currents, but had no effect on direct activation by PMA [91].

CaMKII regulates TRPV1 activity through the phosphorylation of two residues: **S502** and **T704**[46,91].

Phe mutations of the hTRPV1 **Y195**, **Y199**, **Y375**, **Y383** and **Y402** did not diminish Src kinase dependent phosphorylation. But when **Y200** (corresponding to **Y199** in rTRPV1) was mutated, Src-dependent, NGF-induced Tyr phosphorylation was completely abolished [211].

A glycosylation site (**N604**) was identified by Wirkner et al. [172,212].

### Mutations affecting divalent cations

Site-specific analysis has shown that substitutions of **D646** or **Y671** in the pore domain can reduce the permeability of divalent cations [176,197] (Figure 1). This cation selectivity is dynamic, not static, and can vary depending on the stimulus duration or agonist concentration. Activation can alter the Ca^2+^ permeability and pore diameter of TRPV1 to allow influx of larger cations. This change in permeability is mediated by amino acid residues in TM6. Within this domain, **L681** can regulate the permeability of large cations, while **Y671** gates the access of smaller cations [169,178]. Our group discovered a blocking effect of divalent heavy metal cations and especially of Co^2+^ on rTRPV1 [213]. The effects of the cations were evaluated in rTRPV1 containing mutations reported earlier in context of proton activation and tarantula toxin effect. The Co^2+^ sensitivity was slightly reduced in the **D646N** mutant. The **Y627W**, **N628W** and **E651W** mutants displayed little or no difference as compared with the wild type channel [213].

### Mutations of structural involvement

Deletion studies have shown that the C-terminal TRP domain (**E684-R721**) regulates the formation of functional channel tetramers [214]. Removal of this region prevents the oligomerization into stable TRPV1 heteromers [169].

### Mutations causing heightened base activity

Constitutively active TRPV1 mutants might harbor deficits in some aspect of channel activation, and a comprehensive list of such mutations could provide valuable information about the location of the channel gate.

Jordt et al. [43] showed that HEK293 cells expressing **E600Q** TRPV1 channels showed markedly reduced viability. Replacement of this Glu residue with Gln (**E600Q**) or a positively charged amino acid (**E600K**) resulted in a significant level of cell death in HEK293 cells expressing these mutant channels, over their heightened activity under normal culture conditions, whereas substitution with an acidic residue (**E600D**) was not deleterious (Figure 1). This observation suggested that a decrease in negative charge at the **E600** site favours channel activation. The **E600K** mutant showed a most dramatic sensitization phenotype. These channels were already activated at temperature thresholds (30–32°C) well below normal (43°C), resembling the heat sensitivity of wild type channels at pH 6.3 [43].

By genetic screening of a randomly generated population of TRPV1 mutants, Myers et al. [177] demonstrated that mutations within the pore helix domain dramatically increased basal channel activity and responsiveness to chemical and thermal stimuli. The screening for gain of function mutations revealed a total of 30 unique mutations at 25 amino acid positions: **C73S**, **K160E**, **S343R**, **A350T**, **Q561R**, **M581T**, **M609T**, **F640L**, **N652D**, **L673I**, **E684G**, **E684V**, **K710R**, **F742S**, **W787R** and **L796P** caused strong toxicity when expressed in *Saccharomyces cerevisiae*, whereas other gain of function mutations **I75F**, **I76T**, **V78G**, **K155E**, **H166R**, **N310D**, **S343G**, **I352N**, **I352T**, **Q560R**, **M609V**, **I689V**, **L792P** and **L796V** elicited weak toxicity to the cells.

Some mutants displayed large basal currents at pH 7.4, which could be blocked by RuRed. The authors classified mutants as constitutively active when the ratio of the basal inward currents and the CAPS-elicited currents exceeded 0.15 (I_basal_/I_cap_ ≥ 15%). The mutants **K155E**, **K160E**, **M581T** and **F640L** achieved a rank constitutively active.

**F640L** displayed the strongest basal channel activation, and conferred substantial toxicity when expressed in HEK293 cells, characterized by necrotic morphology similar to that observed in cells expressing wild type TRPV1 after prolonged exposure to CAPS. Inclusion of RuRed (3 mM) in the culture medium significantly attenuated the death of **F640L**-expressing cells.

Inside-out patches from the **F640L** mutant displayed large basal currents with a substantial inward component but CAPS at saturating concentrations elicited currents of similar magnitude to those evoked in patches containing wild type channels.

Myers et al. found no significant difference in either the single-channel conductance or the relative permeabilities for Na^+^, K^+^, and Ca^2+^ ions when comparing wild type and **F640L** mutant channels, showing that the **F640L** mutation affects gating rather than permeation properties. Consistent with a hypersensitive gating mechanism, **F640L** mutant displayed a 35-fold leftward shift in the CAPS dose-response curve compared to the wild type receptor, the basal current however, was suppressed by CapZ demonstrating that the high constitutive activity is not due to an inability of the channel to close. Consequently, the gating machinery seems to remain intact in the **F640L** mutant, but the equilibrium appears to be shifted to favor the open state. **F640L** mutation enhances sensitivity to heat and CAPS by shifting the stimulus- response relationships of the channel leftward while also decreasing apparent cooperativity of gating.

To fully explore the structural requirements at position **F640** a codon randomization was performed. Most substitutions at this position, particularly those of a hydrophilic nature, weakened or abolished channel activity. **F640K**, **F640Q**, **F640S**, **F640H**, **F640Y** weakly, whilst **F640C**, **F640E**, **F640G**, **F640N**, **F640R**, **F640D**, **F640P** strongly decreased channel function. Furthermore, **F640A** and **F640T** represented an intermediate phenotype between wild type and weak loss of function. In contrast, several hydrophobic amino acids supported wild type functionality (**F640M**, **F640V**, **F640W**), except for Leu and Ile, which produced constitutively active channels (**F640L**, **F640I**). Thus this codon randomization illustrated that most hydrophobic substitutions at **F640** produced functional channels, whereas two small hydrophobic residues supported constitutive channel activity, suggesting that **F640** is buried in a non-polar environment. Myers et al. uncovered nine additional substitutions conferring a toxic phenotype in yeast (**N625D**, **N628D**, **C634S**, **T641S**, **T650S**, **K656E**, **K656Q**, **V658A** and **F659Y**). These mutants were unable to grow on replica plates containing CAPS but no RuRed. Of these, two (**T641S** and **T650S**) showed high basal activity. Interestingly, **T641S** (and, to a lesser extent, **T650S**) mutants displayed large constitutive channel activation with relative insensitivity to pH 6.4 [177].

### Mutations having deleterious effects on channel function

The E610Q mutants exhibited relatively small responses to any stimuli (CAPS, heat or pH), suggesting that this substitution had deleterious effects on the channel function at large [43]. The function of the channel appeared quite sensitive to perturbations at the position **V538** (Figure 1). Substitution with Gly (**V538G**) resulted in non-functional channels [192]. Susankova et al. [175] mutated and examined the residues **Y666**-**G683** of TRPV1. **Y666A** and **N676A** showed no CAPS-, pH- or heat-evoked activity and exhibited no currents in response to 47°C and 30 μM CAPS applied together. They were determined to be non-functional mutants, suggesting that these two residues within the inner-pore region of rTRPV1 are critical for channel functionality. Boukalova et al. [179] identified mutations **Y554A**, **Y555S** and **E570L** and two charge-reversing mutations, **R557E** and **R579E**, that led to a complete loss of function. In these mutants, 10 μM CAPS neither induced measurable currents at a holding potential of −70 mV, nor affected voltage-dependent (up to +200 mV) or heat-dependent currents (up to 48°C). The **Y554F** and **Y555F** mutations produced fully functional channels, indicating that aromatic side-chains are required at these positions.

### Mutations providing structural information of the channel without having phenotype

Ryu et al. investigated mutations of other residues (**D601-S611**, **Y627-F640** and **D646-E651**) that had minimal consequences, leaving CAPS and low pH responses mostly intact. These included radical perturbations such as the charge mutations **N628R** and **S632D**, suggesting that these residues are probably exposed to the aqueous phase. Such an arrangement would be consistent with their positions in a helix, which renders **T633** facing away from the aqueous phase, making it accessible to interaction with other residues [192]. The function of the channel appeared quite sensitive to perturbations at the position **538** (**V538L**, **V538A**, **V538G**, **V538I**, **V538T**). Even the relatively conservative substitution with Ala abrogated the low pH currents and also reduced the CAPS activity. Further reduction of the size of the side-chain with a substitution by Gly resulted in non-functional channels. It appeared that the local structure of the channel lacks rigidity to tolerate a smaller side-chain at this position. However, the size of the side-chain was not the only factor determining the function of the residue. Replacement by Ile, which has the same volume as Leu, recovered a small, but significant portion of the low pH activity. The data appeared to consist of two populations, one similar to the wild type and the other to **V538L**, as if the local structure of the channel existed in some metastates. Finally, Thr substitution, which preserved the side-chain size, did not confer the wild type responses either. Together, these data indicate that multiple aspects of the side-chain, including both volume and steric hindrance are important for the function of the residue [192].

### Deletion mutants

An N-terminus deletion mutant by Jung et al. [16], Δ^1–109^, which elicited I_cap_, retained specific binding for [^3^H]RTX. In contrast, cells expressing Δ^1–114^, which failed to show I_cap_, completely lost their specific binding to [^3^H]RTX. Similarly, deletion mutants at the C terminus, such as Δ^762–838^ and Δ^763–838^, which displayed smaller I_cap_, bound [^3^H]RTX similarly to the wild type. In contrast, Δ^761–838^, which failed to elicit I_cap_, had no ability to specifically bind [^3^H]RTX. Deletion mutants that lacked one of these two regions lost current sensitivity to CAPS and the ability to bind ligands. The two regions in the vicinity of **R114** and **E761** are critical for ligand binding and that the loss of these regions abrogates vanilloid activation. A deletion as small as a single amino acid affects the ligand binding. Δ^114^ and Δ^761^ failed to elicit CAPS-sensitive inward currents. Furthermore, the mutants did not show specific binding to [^3^H]RTX. Both mutants elicited whole-cell currents when activated by heat at ~46°C, but both mutants failed to respond to acid (pH 5.5) [16].

Deletion of a Thr residue in TM4 (Δ^T550^) reduced CAPS sensitivity [171] (Figure 1).

To investigate the contribution of the pore turret to the TRPV1 function, Cui et al. [190] generated a series of turret deletion mutations by deleting the first 10 (Δ^G603-S612^), 17 (Δ^G603-G619^), or all 24 amino acids (Δ^G603- N626^), and also deleted seven amino acids from the C-terminal end of the turret (Δ^S620-N626^).

Δ^G603-S612^ mutant channel was found to be functional, and exhibited nearly identical CAPS sensitivity to that of the wild type. As CAPS exhibited very similar potency and efficacy in activating wild type and Δ^G603-S612^ channels, this partial turret deletion does not seem to directly affect CAPS activation.

Δ^G603-S612^ reduced the heat response of the channel, which was not a result of slow gating kinetics, as lengthening the heat pulse did not yield any increase in the current amplitude, heat becomes a less effective activator for the Δ^G603-S612^ mutant channel.

Cui et al. found that, the turret deletion mutations Δ^G603-G619^ and Δ^G603-N626^ completely disrupted channel function; no current could be detected from channel-expressing cells challenged with either 10 μM CAPS or high temperatures up to 50°C despite the normal cellular distribution of the mutant channels similar to that of the wild type TRPV1 [190].

## Competing interests

The authors declare that they have no competing interests.

## Authors’ contributions

ZW designed the study prepared the manuscript the figure and the table. AB, FÖ, KJ and CV have been involved in drafting and revising the manuscript. GD, GS and ZO have been involved in revising the manuscript and in the interpretation of data. All authors read and approved the final manuscript.

## Supplementary Material

Additional file 1 Summary of the mutated sites of rTRPV1. Click here for file

## References

[B1] Carlson AE Westenbroek RE Quill T Ren D Clapham DE Hille B Garbers DL Babcock DF CatSper1 required for evoked Ca2+ entry and control of flagellar function in sperm Proc Natl Acad Sci U S A 2003 100 14864 14868 10.1073/pnas.2536658100 14657352 PMC299831

[B2] Cosens DJ Manning A Abnormal electroretinogram from a Drosophila mutant Nature 1969 224 285 287 10.1038/224285a0 5344615

[B3] O'Neill J Brock C Olesen AE Andresen T Nilsson M Dickenson AH Unravelling the mystery of capsaicin: a tool to understand and treat pain Pharmacol Rev 2012 64 939 971 10.1124/pr.112.006163 23023032 PMC3462993

[B4] Venkatachalam K Montell C TRP channels Annu Rev Biochem 2007 76 387 417 10.1146/annurev.biochem.75.103004.142819 17579562 PMC4196875

[B5] Owsianik G Talavera K Voets T Nilius B Permeation and selectivity of TRP channels Annu Rev Physiol 2006 68 685 717 10.1146/annurev.physiol.68.040204.101406 16460288

[B6] Li M Yu Y Yang J Structural biology of TRP channels Adv Exp Med Biol 2011 704 1 23 21290287 10.1007/978-94-007-0265-3_1 PMC4356010

[B7] Landry Y Gies JP Drugs and their molecular targets: an updated overview Fundam Clin Pharmacol 2008 22 1 18 18251718 10.1111/j.1472-8206.2007.00548.x

[B8] Okuhara DY Hsia AY Xie M Transient receptor potential channels as drug targets Expert Opin Ther Targets 2007 11 391 401 10.1517/14728222.11.3.391 17298296

[B9] Szallasi A Cortright DN Blum CA Eid SR The vanilloid receptor TRPV1: 10 years from channel cloning to antagonist proof-of-concept Nat Rev Drug Discov 2007 6 357 372 10.1038/nrd2280 17464295

[B10] Nilius B Vennekens R Gomtsyan A, Faltynek CR Vanilloid Receptor TRPV1 in Drug Discovery: Targeting Pain and Other Pathological Disorders 2010 Hoboken, NJ, USA: John Wiley & Sons, Inc

[B11] Xu SZ Zeng F Boulay G Grimm C Harteneck C Beech DJ Block of TRPC5 channels by 2-aminoethoxydiphenyl borate: a differential, extracellular and voltage-dependent effect Br J Pharmacol 2005 145 405 414 15806115 10.1038/sj.bjp.0706197 PMC1576154

[B12] Jin X Touhey J Gaudet R Structure of the N-terminal ankyrin repeat domain of the TRPV2 ion channel J Biol Chem 2006 281 25006 25010 10.1074/jbc.C600153200 16809337

[B13] McCleverty CJ Koesema E Patapoutian A Lesley SA Kreusch A Crystal structure of the human TRPV2 channel ankyrin repeat domain Protein Sci 2006 15 2201 2206 10.1110/ps.062357206 16882997 PMC2242602

[B14] Lishko PV Procko E Jin X Phelps CB Gaudet R The ankyrin repeats of TRPV1 bind multiple ligands and modulate channel sensitivity Neuron 2007 54 905 918 10.1016/j.neuron.2007.05.027 17582331

[B15] Hellwig N Albrecht N Harteneck C Schultz G Schaefer M Homo- and heteromeric assembly of TRPV channel subunits J Cell Sci 2005 118 917 928 10.1242/jcs.01675 15713749

[B16] Jung J Lee SY Hwang SW Cho H Shin J Kang YS Kim S Oh U Agonist recognition sites in the cytosolic tails of vanilloid receptor 1 J Biol Chem 2002 277 44448 44454 10.1074/jbc.M207103200 12228246

[B17] Mosavi LK Cammett TJ Desrosiers DC Peng ZY The ankyrin repeat as molecular architecture for protein recognition Protein Sci 2004 13 1435 1448 10.1110/ps.03554604 15152081 PMC2279977

[B18] Sedgwick SG Smerdon SJ The ankyrin repeat: a diversity of interactions on a common structural framework Trends Biochem Sci 1999 24 311 316 10.1016/S0968-0004(99)01426-7 10431175

[B19] Vennekens R Hoenderop JG Prenen J Stuiver M Willems PH Droogmans G Nilius B Bindels RJ Permeation and gating properties of the novel epithelial Ca(2+) channel J Biol Chem 2000 275 3963 3969 10.1074/jbc.275.6.3963 10660551

[B20] Yue L Peng JB Hediger MA Clapham DE CaT1 manifests the pore properties of the calcium-release-activated calcium channel Nature 2001 410 705 709 10.1038/35070596 11287959

[B21] Dhaka A Viswanath V Patapoutian A Trp ion channels and temperature sensation Annu Rev Neurosci 2006 29 135 161 10.1146/annurev.neuro.29.051605.112958 16776582

[B22] Lawson JJ McIlwrath SL Woodbury CJ Davis BM Koerber HR TRPV1 unlike TRPV2 is restricted to a subset of mechanically insensitive cutaneous nociceptors responding to heat J Pain 2008 9 298 308 10.1016/j.jpain.2007.12.001 18226966 PMC2372162

[B23] Xu H Delling M Jun JC Clapham DE Oregano, thyme and clove-derived flavors and skin sensitizers activate specific TRP channels Nat Neurosci 2006 9 628 635 10.1038/nn1692 16617338

[B24] Moqrich A Hwang SW Earley TJ Petrus MJ Murray AN Spencer KS Andahazy M Story GM Patapoutian A Impaired thermosensation in mice lacking TRPV3, a heat and camphor sensor in the skin Science 2005 307 1468 1472 10.1126/science.1108609 15746429

[B25] Nilius B Owsianik G Voets T Peters JA Transient receptor potential cation channels in disease Physiol Rev 2007 87 165 217 10.1152/physrev.00021.2006 17237345

[B26] Hoenderop JG van der Kemp AW Hartog A van de Graaf SF van Os CH Willems PH Bindels RJ Molecular identification of the apical Ca2+ channel in 1, 25-dihydroxyvitamin D3-responsive epithelia J Biol Chem 1999 274 8375 8378 10.1074/jbc.274.13.8375 10085067

[B27] Peng JB Chen XZ Berger UV Vassilev PM Tsukaguchi H Brown EM Hediger MA Molecular cloning and characterization of a channel-like transporter mediating intestinal calcium absorption J Biol Chem 1999 274 22739 22746 10.1074/jbc.274.32.22739 10428857

[B28] Caterina MJ Schumacher MA Tominaga M Rosen TA Levine JD Julius D The capsaicin receptor: a heat-activated ion channel in the pain pathway Nature 1997 389 816 824 10.1038/39807 9349813

[B29] Kedei N Szabo T Lile JD Treanor JJ Olah Z Iadarola MJ Blumberg PM Analysis of the native quaternary structure of vanilloid receptor 1 J Biol Chem 2001 276 28613 28619 10.1074/jbc.M103272200 11358970

[B30] Moiseenkova-Bell VY Stanciu LA Serysheva, II, Tobe BJ, Wensel TG: Structure of TRPV1 channel revealed by electron cryomicroscopy Proc Natl Acad Sci U S A 2008 105 7451 7455 10.1073/pnas.0711835105 18490661 PMC2396679

[B31] Vriens J Appendino G Nilius B Pharmacology of vanilloid transient receptor potential cation channels Mol Pharmacol 2009 75 1262 1279 10.1124/mol.109.055624 19297520

[B32] Szallasi A Blumberg PM Resiniferatoxin, a phorbol-related diterpene, acts as an ultrapotent analog of capsaicin, the irritant constituent in red pepper Neuroscience 1989 30 515 520 10.1016/0306-4522(89)90269-8 2747924

[B33] Latorre R Brauchi S Orta G Zaelzer C Vargas G ThermoTRP channels as modular proteins with allosteric gating Cell Calcium 2007 42 427 438 10.1016/j.ceca.2007.04.004 17499848

[B34] Szallasi A The vanilloid (capsaicin) receptor: receptor types and species differences Gen Pharmacol 1994 25 223 243 10.1016/0306-3623(94)90049-3 8026721

[B35] Witte DG Cassar SC Masters JN Esbenshade T Hancock AA Use of a fluorescent imaging plate reader–based calcium assay to assess pharmacological differences between the human and rat vanilloid receptor J Biomol Screen 2002 7 466 475 10.1177/108705702237679 14599363

[B36] Culshaw AJ Bevan S Christiansen M Copp P Davis A Davis C Dyson A Dziadulewicz EK Edwards L Eggelte H Identification and biological characterization of 6-aryl-7-isopropylquinazolinones as novel TRPV1 antagonists that are effective in models of chronic pain J Med Chem 2006 49 471 474 10.1021/jm051058x 16420034

[B37] Pearce LV Petukhov PA Szabo T Kedei N Bizik F Kozikowski AP Blumberg PM Evodiamine functions as an agonist for the vanilloid receptor TRPV1 Org Biomol Chem 2004 2 2281 2286 10.1039/b404506h 15305207

[B38] Xu S Cheng Y Keast JR Osborne PB 17beta-estradiol activates estrogen receptor beta-signalling and inhibits transient receptor potential vanilloid receptor 1 activation by capsaicin in adult rat nociceptor neurons Endocrinology 2008 149 5540 5548 10.1210/en.2008-0278 18617618 PMC2584594

[B39] De Petrocellis L Bisogno T Davis JB Pertwee RG Di Marzo V Overlap between the ligand recognition properties of the anandamide transporter and the VR1 vanilloid receptor: inhibitors of anandamide uptake with negligible capsaicin-like activity FEBS Lett 2000 483 52 56 10.1016/S0014-5793(00)02082-2 11033355

[B40] Ross RA Anandamide and vanilloid TRPV1 receptors Br J Pharmacol 2003 140 790 801 10.1038/sj.bjp.0705467 14517174 PMC1574087

[B41] Ahern GP Activation of TRPV1 by the satiety factor oleoylethanolamide J Biol Chem 2003 278 30429 30434 10.1074/jbc.M305051200 12761211

[B42] Hwang SW Cho H Kwak J Lee SY Kang CJ Jung J Cho S Min KH Suh YG Kim D Oh U Direct activation of capsaicin receptors by products of lipoxygenases: endogenous capsaicin-like substances Proc Natl Acad Sci U S A 2000 97 6155 6160 10.1073/pnas.97.11.6155 10823958 PMC18574

[B43] Jordt SE Tominaga M Julius D Acid potentiation of the capsaicin receptor determined by a key extracellular site Proc Natl Acad Sci U S A 2000 97 8134 8139 10.1073/pnas.100129497 10859346 PMC16682

[B44] Szallasi A Blumberg PM Vanilloid (Capsaicin) receptors and mechanisms Pharmacol Rev 1999 51 159 212 10353985

[B45] Yang BH Piao ZG Kim YB Lee CH Lee JK Park K Kim JS Oh SB Activation of vanilloid receptor 1 (VR1) by eugenol J Dent Res 2003 82 781 785 10.1177/154405910308201004 14514756

[B46] Jara-Oseguera A Simon SA Rosenbaum T TRPV1: on the road to pain relief Curr Mol Pharmacol 2008 1 255 269 20021438 10.2174/1874467210801030255 PMC2802457

[B47] Premkumar LS Qi ZH Van Buren J Raisinghani M Enhancement of potency and efficacy of NADA by PKC-mediated phosphorylation of vanilloid receptor J Neurophysiol 2004 91 1442 1449 14973326 10.1152/jn.00745.2003

[B48] Price TJ Patwardhan A Akopian AN Hargreaves KM Flores CM Modulation of trigeminal sensory neuron activity by the dual cannabinoid-vanilloid agonists anandamide, N-arachidonoyl-dopamine and arachidonyl-2-chloroethylamide Br J Pharmacol 2004 141 1118 1130 10.1038/sj.bjp.0705711 15006899 PMC1574881

[B49] Suh YG Oh U Activation and activators of TRPV1 and their pharmaceutical implication Curr Pharm Des 2005 11 2687 2698 10.2174/1381612054546789 16101449

[B50] Liu L Simon SA Similarities and differences in the currents activated by capsaicin, piperine, and zingerone in rat trigeminal ganglion cells J Neurophysiol 1996 76 1858 1869 8890298 10.1152/jn.1996.76.3.1858

[B51] McNamara FN Randall A Gunthorpe MJ Effects of piperine, the pungent component of black pepper, at the human vanilloid receptor (TRPV1) Br J Pharmacol 2005 144 781 790 10.1038/sj.bjp.0706040 15685214 PMC1576058

[B52] Dedov VN Tran VH Duke CC Connor M Christie MJ Mandadi S Roufogalis BD Gingerols: a novel class of vanilloid receptor (VR1) agonists Br J Pharmacol 2002 137 793 798 10.1038/sj.bjp.0704925 12411409 PMC1573550

[B53] Iwasaki Y Morita A Iwasawa T Kobata K Sekiwa Y Morimitsu Y Kubota K Watanabe T A nonpungent component of steamed ginger–[10]-shogaol–increases adrenaline secretion via the activation of TRPV1 Nutr Neurosci 2006 9 169 178 10.1080/10284150600955164 17176640

[B54] Jung J Hwang SW Kwak J Lee SY Kang CJ Kim WB Kim D Oh U Capsaicin binds to the intracellular domain of the capsaicin-activated ion channel J Neurosci 1999 19 529 538 9880573 10.1523/JNEUROSCI.19-02-00529.1999 PMC6782213

[B55] Bootman MD Collins TJ Mackenzie L Roderick HL Berridge MJ Peppiatt CM 2-aminoethoxydiphenyl borate (2-APB) is a reliable blocker of store-operated Ca2+ entry but an inconsistent inhibitor of InsP3-induced Ca2+ release FASEB J 2002 16 1145 1150 10.1096/fj.02-0037rev 12153982

[B56] Bautista DM Jordt SE Nikai T Tsuruda PR Read AJ Poblete J Yamoah EN Basbaum AI Julius D TRPA1 mediates the inflammatory actions of environmental irritants and proalgesic agents Cell 2006 124 1269 1282 10.1016/j.cell.2006.02.023 16564016

[B57] Macpherson LJ Geierstanger BH Viswanath V Bandell M Eid SR Hwang S Patapoutian A The pungency of garlic: activation of TRPA1 and TRPV1 in response to allicin Curr Biol 2005 15 929 934 10.1016/j.cub.2005.04.018 15916949

[B58] Everaerts W Gees M Alpizar YA Farre R Leten C Apetrei A Dewachter I van Leuven F Vennekens R De Ridder D The capsaicin receptor TRPV1 is a crucial mediator of the noxious effects of mustard oil Curr Biol 2011 21 316 321 10.1016/j.cub.2011.01.031 21315593

[B59] Mori N Kawabata F Matsumura S Hosokawa H Kobayashi S Inoue K Fushiki T Intragastric administration of allyl isothiocyanate increases carbohydrate oxidation via TRPV1 but not TRPA1 in mice Am J Physiol Regul Integr Comp Physiol 2011 300 R1494 1505 10.1152/ajpregu.00645.2009 21430076

[B60] Bandell M Story GM Hwang SW Viswanath V Eid SR Petrus MJ Earley TJ Patapoutian A Noxious cold ion channel TRPA1 is activated by pungent compounds and bradykinin Neuron 2004 41 849 857 10.1016/S0896-6273(04)00150-3 15046718

[B61] Story GM Peier AM Reeve AJ Eid SR Mosbacher J Hricik TR Earley TJ Hergarden AC Andersson DA Hwang SW ANKTM1, a TRP-like channel expressed in nociceptive neurons, is activated by cold temperatures Cell 2003 112 819 829 10.1016/S0092-8674(03)00158-2 12654248

[B62] Bautista DM Movahed P Hinman A Axelsson HE Sterner O Hogestatt ED Julius D Jordt SE Zygmunt PM Pungent products from garlic activate the sensory ion channel TRPA1 Proc Natl Acad Sci U S A 2005 102 12248 12252 10.1073/pnas.0505356102 16103371 PMC1189336

[B63] Pecze L Pelsoczi P Kecskes M Winter Z Papp A Kaszas K Letoha T Vizler C Olah Z Resiniferatoxin mediated ablation of TRPV1+ neurons removes TRPA1 as well Can J Neurol Sci 2009 36 234 241 19378721 10.1017/s0317167100006600

[B64] Szolcsanyi J Sandor Z Multisteric TRPV1 nocisensor: a target for analgesics Trends Pharmacol Sci 2012 33 646 655 10.1016/j.tips.2012.09.002 23068431

[B65] Dray A Bettaney J Forster P Resiniferatoxin, a potent capsaicin-like stimulator of peripheral nociceptors in the neonatal rat tail in vitro Br J Pharmacol 1990 99 323 326 10.1111/j.1476-5381.1990.tb14702.x 2328397 PMC1917382

[B66] Docherty RJ Yeats JC Piper AS Capsazepine block of voltage-activated calcium channels in adult rat dorsal root ganglion neurones in culture Br J Pharmacol 1997 121 1461 1467 10.1038/sj.bjp.0701272 9257928 PMC1564831

[B67] Liu L Simon SA Capsazepine, a vanilloid receptor antagonist, inhibits nicotinic acetylcholine receptors in rat trigeminal ganglia Neurosci Lett 1997 228 29 32 10.1016/S0304-3940(97)00358-3 9197280

[B68] Xia R Dekermendjian K Lullau E Dekker N TRPV1: a therapy target that attracts the pharmaceutical interests Adv Exp Med Biol 2011 704 637 665 10.1007/978-94-007-0265-3_34 21290320

[B69] Seabrook GR Sutton KG Jarolimek W Hollingworth GJ Teague S Webb J Clark N Boyce S Kerby J Ali Z Functional properties of the high-affinity TRPV1 (VR1) vanilloid receptor antagonist (4-hydroxy-5-iodo-3-methoxyphenylacetate ester) iodo-resiniferatoxin J Pharmacol Exp Ther 2002 303 1052 1060 10.1124/jpet.102.040394 12438527

[B70] Planells-Cases R Aracil A Merino JM Gallar J Perez-Paya E Belmonte C Gonzalez-Ros JM Ferrer-Montiel AV Arginine-rich peptides are blockers of VR-1 channels with analgesic activity FEBS Lett 2000 481 131 136 10.1016/S0014-5793(00)01982-7 10996311

[B71] Himmel HM Kiss T Borvendeg SJ Gillen C Illes P The arginine-rich hexapeptide R4W2 is a stereoselective antagonist at the vanilloid receptor 1: a Ca2+ imaging study in adult rat dorsal root ganglion neurons J Pharmacol Exp Ther 2002 301 981 986 10.1124/jpet.301.3.981 12023528

[B72] Gavva NR Body-temperature maintenance as the predominant function of the vanilloid receptor TRPV1 Trends Pharmacol Sci 2008 29 550 557 10.1016/j.tips.2008.08.003 18805596

[B73] Gavva NR Bannon AW Surapaneni S Hovland DN Jr Lehto SG Gore A Juan T Deng H Han B Klionsky L The vanilloid receptor TRPV1 is tonically activated in vivo and involved in body temperature regulation J Neurosci 2007 27 3366 3374 10.1523/JNEUROSCI.4833-06.2007 17392452 PMC6672109

[B74] Gavva NR Treanor JJ Garami A Fang L Surapaneni S Akrami A Alvarez F Bak A Darling M Gore A Pharmacological blockade of the vanilloid receptor TRPV1 elicits marked hyperthermia in humans Pain 2008 136 202 210 10.1016/j.pain.2008.01.024 18337008

[B75] Wang S Poon K Oswald RE Chuang HH Distinct modulations of human capsaicin receptor by protons and magnesium through different domains J Biol Chem 2010 285 11547 11556 10.1074/jbc.M109.058727 20145248 PMC2857033

[B76] Voets T Droogmans G Wissenbach U Janssens A Flockerzi V Nilius B The principle of temperature-dependent gating in cold- and heat-sensitive TRP channels Nature 2004 430 748 754 10.1038/nature02732 15306801

[B77] Ma W Quirion R Inflammatory mediators modulating the transient receptor potential vanilloid 1 receptor: therapeutic targets to treat inflammatory and neuropathic pain Expert Opin Ther Targets 2007 11 307 320 10.1517/14728222.11.3.307 17298290

[B78] Rosenbaum T Simon SA Liedtke WB, Heller S TRPV1 Receptors and Signal Transduction TRP Ion Channel Function in Sensory Transduction and Cellular Signaling Cascades 2007 Boca Raton (FL): CRC Press Chapter 5. Available from: http://www.ncbi.nlm.nih.gov/books/NBK5260/21204486

[B79] Tominaga M Caterina MJ Malmberg AB Rosen TA Gilbert H Skinner K Raumann BE Basbaum AI Julius D The cloned capsaicin receptor integrates multiple pain-producing stimuli Neuron 1998 21 531 543 10.1016/S0896-6273(00)80564-4 9768840

[B80] Olah Z Szabo T Karai L Hough C Fields RD Caudle RM Blumberg PM Iadarola MJ Ligand-induced dynamic membrane changes and cell deletion conferred by vanilloid receptor 1 J Biol Chem 2001 276 11021 11030 10.1074/jbc.M008392200 11124944

[B81] Sprague J Harrison C Rowbotham DJ Smart D Lambert DG Temperature-dependent activation of recombinant rat vanilloid VR1 receptors expressed in HEK293 cells by capsaicin and anandamide Eur J Pharmacol 2001 423 121 125 10.1016/S0014-2999(01)01123-2 11448475

[B82] De Petrocellis L Harrison S Bisogno T Tognetto M Brandi I Smith GD Creminon C Davis JB Geppetti P Di Marzo V The vanilloid receptor (VR1)-mediated effects of anandamide are potently enhanced by the cAMP-dependent protein kinase J Neurochem 2001 77 1660 1663 10.1046/j.1471-4159.2001.00406.x 11413249

[B83] Rathee PK Distler C Obreja O Neuhuber W Wang GK Wang SY Nau C Kress M PKA/AKAP/VR-1 module: A common link of Gs-mediated signaling to thermal hyperalgesia J Neurosci 2002 22 4740 4745 12040081 10.1523/JNEUROSCI.22-11-04740.2002 PMC6758778

[B84] Vlachova V Teisinger J Susankova K Lyfenko A Ettrich R Vyklicky L Functional role of C-terminal cytoplasmic tail of rat vanilloid receptor 1 J Neurosci 2003 23 1340 1350 12598622 10.1523/JNEUROSCI.23-04-01340.2003 PMC6742269

[B85] Bhave G Hu HJ Glauner KS Zhu W Wang H Brasier DJ Oxford GS Gereau RWt: Protein kinase C phosphorylation sensitizes but does not activate the capsaicin receptor transient receptor potential vanilloid 1 (TRPV1) Proc Natl Acad Sci U S A 2003 100 12480 12485 10.1073/pnas.2032100100 14523239 PMC218783

[B86] Zhang X Du XN Zhang GH Jia ZF Chen XJ Huang DY Liu BY Zhang HL Agonist-dependent potentiation of vanilloid receptor transient receptor potential vanilloid type 1 function by stilbene derivatives Mol Pharmacol 2012 81 689 700 10.1124/mol.111.076000 22328719

[B87] Ohta T Imagawa T Ito S Novel agonistic action of mustard oil on recombinant and endogenous porcine transient receptor potential V1 (pTRPV1) channels Biochem Pharmacol 2007 73 1646 1656 10.1016/j.bcp.2007.01.029 17328867

[B88] Czaja K Burns GA Ritter RC Capsaicin-induced neuronal death and proliferation of the primary sensory neurons located in the nodose ganglia of adult rats Neuroscience 2008 154 621 630 10.1016/j.neuroscience.2008.03.055 18456414 PMC2527584

[B89] Olah Z Karai L Iadarola MJ Protein kinase C(alpha) is required for vanilloid receptor 1 activation. Evidence for multiple signaling pathways J Biol Chem 2002 277 35752 35759 10.1074/jbc.M201551200 12095983

[B90] Wang Y Kedei N Wang M Wang QJ Huppler AR Toth A Tran R Blumberg PM Interaction between protein kinase Cmu and the vanilloid receptor type 1 J Biol Chem 2004 279 53674 53682 10.1074/jbc.M410331200 15471852

[B91] Jung J Shin JS Lee SY Hwang SW Koo J Cho H Oh U Phosphorylation of vanilloid receptor 1 by Ca2+/calmodulin-dependent kinase II regulates its vanilloid binding J Biol Chem 2004 279 7048 7054 14630912 10.1074/jbc.M311448200

[B92] Zhou HY Zhang HM Chen SR Pan HL Increased nociceptive input rapidly modulates spinal GABAergic transmission through endogenously released glutamate J Neurophysiol 2007 97 871 882 10.1152/jn.00964.2006 17108089

[B93] Docherty RJ Yeats JC Bevan S Boddeke HW Inhibition of calcineurin inhibits the desensitization of capsaicin-evoked currents in cultured dorsal root ganglion neurones from adult rats Pflugers Arch 1996 431 828 837 8927498 10.1007/s004240050074

[B94] Koplas PA Rosenberg RL Oxford GS The role of calcium in the desensitization of capsaicin responses in rat dorsal root ganglion neurons J Neurosci 1997 17 3525 3537 9133377 10.1523/JNEUROSCI.17-10-03525.1997 PMC6573672

[B95] Prescott ED Julius D A modular PIP2 binding site as a determinant of capsaicin receptor sensitivity Science 2003 300 1284 1288 10.1126/science.1083646 12764195

[B96] Liu B Zhang C Qin F Functional recovery from desensitization of vanilloid receptor TRPV1 requires resynthesis of phosphatidylinositol 4,5-bisphosphate J Neurosci 2005 25 4835 4843 10.1523/JNEUROSCI.1296-05.2005 15888659 PMC6724779

[B97] Stein AT Ufret-Vincenty CA Hua L Santana LF Gordon SE Phosphoinositide 3-kinase binds to TRPV1 and mediates NGF-stimulated TRPV1 trafficking to the plasma membrane J Gen Physiol 2006 128 509 522 10.1085/jgp.200609576 17074976 PMC2151588

[B98] Ufret-Vincenty CA Klein RM Hua L Angueyra J Gordon SE Localization of the PIP2 sensor of TRPV1 ion channels J Biol Chem 2011 286 9688 9698 10.1074/jbc.M110.192526 21224382 PMC3058964

[B99] Yao J Qin F Interaction with phosphoinositides confers adaptation onto the TRPV1 pain receptor PLoS Biol 2009 7 e46 10.1371/journal.pbio.1000046 19243225 PMC3279049

[B100] Levitan I Fang Y Rosenhouse-Dantsker A Romanenko V Cholesterol and ion channels Subcell Biochem 2010 51 509 549 10.1007/978-90-481-8622-8_19 20213557 PMC2895485

[B101] Levitan I Christian AE Tulenko TN Rothblat GH Membrane cholesterol content modulates activation of volume-regulated anion current in bovine endothelial cells J Gen Physiol 2000 115 405 416 10.1085/jgp.115.4.405 10736308 PMC2233759

[B102] Levitan I Cholesterol and Kir channels IUBMB Life 2009 61 781 790 10.1002/iub.192 19548316 PMC2720429

[B103] Liu M Huang W Wu D Priestley JV TRPV1, but not P2X, requires cholesterol for its function and membrane expression in rat nociceptors Eur J Neurosci 2006 24 1 6 10.1111/j.1460-9568.2006.04889.x 16800863

[B104] Szoke E Borzsei R Toth DM Lengl O Helyes Z Sandor Z Szolcsanyi J Effect of lipid raft disruption on TRPV1 receptor activation of trigeminal sensory neurons and transfected cell line Eur J Pharmacol 2010 628 67 74 10.1016/j.ejphar.2009.11.052 19958765

[B105] Santha P Oszlacs O Dux M Dobos I Jancso G Inhibition of glucosylceramide synthase reversibly decreases the capsaicin-induced activation and TRPV1 expression of cultured dorsal root ganglion neurons Pain 2010 150 103 112 10.1016/j.pain.2010.04.006 20427129

[B106] Numazaki M Tominaga T Takeuchi K Murayama N Toyooka H Tominaga M Structural determinant of TRPV1 desensitization interacts with calmodulin Proc Natl Acad Sci U S A 2003 100 8002 8006 10.1073/pnas.1337252100 12808128 PMC164702

[B107] Rosenbaum T Gordon-Shaag A Munari M Gordon SE Ca2+/calmodulin modulates TRPV1 activation by capsaicin J Gen Physiol 2004 123 53 62 14699077 10.1085/jgp.200308906 PMC2217413

[B108] Vyklicky L Novakova-Tousova K Benedikt J Samad A Touska F Vlachova V Calcium-dependent desensitization of vanilloid receptor TRPV1: a mechanism possibly involved in analgesia induced by topical application of capsaicin Physiol Res 2008 57 Suppl 3 S59 68 18481914 10.33549/physiolres.931478

[B109] Tominaga M Wada M Masu M Potentiation of capsaicin receptor activity by metabotropic ATP receptors as a possible mechanism for ATP-evoked pain and hyperalgesia Proc Natl Acad Sci U S A 2001 98 6951 6956 10.1073/pnas.111025298 11371611 PMC34459

[B110] Vellani V Mapplebeck S Moriondo A Davis JB McNaughton PA Protein kinase C activation potentiates gating of the vanilloid receptor VR1 by capsaicin, protons, heat and anandamide J Physiol 2001 534 813 825 10.1111/j.1469-7793.2001.00813.x 11483711 PMC2278732

[B111] Farkas B Bonnekoh B Mahrle G Repeated treatment with dithranol induces a tolerance reaction in keratinocytes in vitro Arch Dermatol Res 1991 283 337 341 10.1007/BF00376624 1929556

[B112] Susankova K Tousova K Vyklicky L Teisinger J Vlachova V Reducing and oxidizing agents sensitize heat-activated vanilloid receptor (TRPV1) current Mol Pharmacol 2006 70 383 394 16614139 10.1124/mol.106.023069

[B113] Cesare P Dekker LV Sardini A Parker PJ McNaughton PA Specific involvement of PKC-epsilon in sensitization of the neuronal response to painful heat Neuron 1999 23 617 624 10.1016/S0896-6273(00)80813-2 10433272

[B114] Premkumar LS Ahern GP Induction of vanilloid receptor channel activity by protein kinase C Nature 2000 408 985 990 10.1038/35050121 11140687

[B115] Chuang HH Prescott ED Kong H Shields S Jordt SE Basbaum AI Chao MV Julius D Bradykinin and nerve growth factor release the capsaicin receptor from PtdIns(4,5)P2-mediated inhibition Nature 2001 411 957 962 10.1038/35082088 11418861

[B116] Bhave G Zhu W Wang H Brasier DJ Oxford GS Gereau RWt: cAMP-dependent protein kinase regulates desensitization of the capsaicin receptor (VR1) by direct phosphorylation Neuron 2002 35 721 731 10.1016/S0896-6273(02)00802-4 12194871

[B117] Shin HJ Gye MH Chung KH Yoo BS Activity of protein kinase C modulates the apoptosis induced by polychlorinated biphenyls in human leukemic HL-60 cells Toxicol Lett 2002 135 25 31 10.1016/S0378-4274(02)00231-X 12243861

[B118] Bevan S Andersson DA TRP channel antagonists for pain–opportunities beyond TRPV1 Curr Opin Investig Drugs 2009 10 655 663 19579171

[B119] Cortright DN Szallasi A TRP channels and pain Curr Pharm Des 2009 15 1736 1749 10.2174/138161209788186308 19442187

[B120] Stucky CL Dubin AE Jeske NA Malin SA McKemy DD Story GM Roles of transient receptor potential channels in pain Brain Res Rev 2009 60 2 23 10.1016/j.brainresrev.2008.12.018 19203589 PMC2683630

[B121] Fernandes ES Russell FA Spina D McDougall JJ Graepel R Gentry C Staniland AA Mountford DM Keeble JE Malcangio M A distinct role for transient receptor potential ankyrin 1, in addition to transient receptor potential vanilloid 1, in tumor necrosis factor alpha-induced inflammatory hyperalgesia and Freund's complete adjuvant-induced monarthritis Arthritis Rheum 2011 63 819 829 10.1002/art.30150 21360511

[B122] Fernandes ES Fernandes MA Keeble JE The functions of TRPA1 and TRPV1: moving away from sensory nerves Br J Pharmacol 2012 166 510 521 10.1111/j.1476-5381.2012.01851.x 22233379 PMC3417484

[B123] Alawi K Keeble J The paradoxical role of the transient receptor potential vanilloid 1 receptor in inflammation Pharmacol Ther 2010 125 181 195 10.1016/j.pharmthera.2009.10.005 19896501

[B124] Dux M Santha P Jancso G The role of chemosensitive afferent nerves and TRP ion channels in the pathomechanism of headaches Pflugers Arch 2012 464 239 248 10.1007/s00424-012-1142-7 22875278

[B125] Jin K Xie L Kim SH Parmentier-Batteur S Sun Y Mao XO Childs J Greenberg DA Defective adult neurogenesis in CB1 cannabinoid receptor knockout mice Mol Pharmacol 2004 66 204 208 10.1124/mol.66.2.204 15266010

[B126] Jancso-Gabor A Szolcsanyi J Jancso N Stimulation and desensitization of the hypothalamic heat-sensitive structures by capsaicin in rats J Physiol 1970 208 449 459 5500735 10.1113/jphysiol.1970.sp009130 PMC1348759

[B127] Jancso G Wollemann M The effect of capsaicin on the adenylate cyclase activity of rat brain Brain Res 1977 123 323 329 10.1016/0006-8993(77)90483-8 191145

[B128] Dib B Effects of intracerebroventricular capsaicin on thermoregulatory behavior in the rat Pharmacol Biochem Behav 1982 16 23 27 10.1016/0091-3057(82)90007-7 7058212

[B129] Steiner AA Turek VF Almeida MC Burmeister JJ Oliveira DL Roberts JL Bannon AW Norman MH Louis JC Treanor JJ Nonthermal activation of transient receptor potential vanilloid-1 channels in abdominal viscera tonically inhibits autonomic cold-defense effectors J Neurosci 2007 27 7459 7468 10.1523/JNEUROSCI.1483-07.2007 17626206 PMC6672610

[B130] Gavva NR Bannon AW Hovland DN Jr Lehto SG Klionsky L Surapaneni S Immke DC Henley C Arik L Bak A Repeated administration of vanilloid receptor TRPV1 antagonists attenuates hyperthermia elicited by TRPV1 blockade J Pharmacol Exp Ther 2007 323 128 137 10.1124/jpet.107.125674 17652633

[B131] Tamayo N Liao H Stec MM Wang X Chakrabarti P Retz D Doherty EM Surapaneni S Tamir R Bannon AW Design and synthesis of peripherally restricted transient receptor potential vanilloid 1 (TRPV1) antagonists J Med Chem 2008 51 2744 2757 10.1021/jm7014638 18386885

[B132] Inoue K Koizumi S Fuziwara S Denda S Denda M Functional vanilloid receptors in cultured normal human epidermal keratinocytes Biochem Biophys Res Commun 2002 291 124 129 10.1006/bbrc.2002.6393 11829471

[B133] Kim SJ Lee SA Yun SJ Kim JK Park JS Jeong HS Lee JH Moon SJ Won YH Expression of vanilloid receptor 1 in cultured fibroblast Exp Dermatol 2006 15 362 367 10.1111/j.0906-6705.2006.00418.x 16630076

[B134] Grone A Fonfara S Baumgartner W Cell type-dependent cytokine expression after canine distemper virus infection Viral Immunol 2002 15 493 505 10.1089/088282402760312368 12479398

[B135] Southall MD Li T Gharibova LS Pei Y Nicol GD Travers JB Activation of epidermal vanilloid receptor-1 induces release of proinflammatory mediators in human keratinocytes J Pharmacol Exp Ther 2003 304 217 222 10.1124/jpet.102.040675 12490594

[B136] Saunders CI Kunde DA Crawford A Geraghty DP Expression of transient receptor potential vanilloid 1 (TRPV1) and 2 (TRPV2) in human peripheral blood Mol Immunol 2007 44 1429 1435 10.1016/j.molimm.2006.04.027 16777226

[B137] Basu S Srivastava P Immunological role of neuronal receptor vanilloid receptor 1 expressed on dendritic cells Proc Natl Acad Sci U S A 2005 102 5120 5125 10.1073/pnas.0407780102 15793000 PMC555601

[B138] Geppetti P Nassini R Materazzi S Benemei S The concept of neurogenic inflammation BJU Int 2008 101 Suppl 3 2 6 18307678 10.1111/j.1464-410X.2008.07493.x

[B139] Earley S Gonzales AL Crnich R Endothelium-dependent cerebral artery dilation mediated by TRPA1 and Ca2+−Activated K+ channels Circ Res 2009 104 987 994 10.1161/CIRCRESAHA.108.189530 19299646 PMC2966339

[B140] Kark T Bagi Z Lizanecz E Pasztor ET Erdei N Czikora A Papp Z Edes I Porszasz R Toth A Tissue-specific regulation of microvascular diameter: opposite functional roles of neuronal and smooth muscle located vanilloid receptor-1 Mol Pharmacol 2008 73 1405 1412 10.1124/mol.107.043323 18256211

[B141] Luo D Zhang YW Peng WJ Peng J Chen QQ Li D Deng HW Li YJ Transient receptor potential vanilloid 1-mediated expression and secretion of endothelial cell-derived calcitonin gene-related peptide Regul Pept 2008 150 66 72 10.1016/j.regpep.2008.05.007 18584893

[B142] Brain SD Williams TJ Tippins JR Morris HR MacIntyre I Calcitonin gene-related peptide is a potent vasodilator Nature 1985 313 54 56 10.1038/313054a0 3917554

[B143] Zygmunt PM Petersson J Andersson DA Chuang H Sorgard M Di Marzo V Julius D Hogestatt ED Vanilloid receptors on sensory nerves mediate the vasodilator action of anandamide Nature 1999 400 452 457 10.1038/22761 10440374

[B144] Keeble JE Brain SD Capsaicin-induced vasoconstriction in the mouse knee joint: a study using TRPV1 knockout mice Neurosci Lett 2006 401 55 58 10.1016/j.neulet.2006.02.083 16584841

[B145] Cavanaugh DJ Chesler AT Jackson AC Sigal YM Yamanaka H Grant R O'Donnell D Nicoll RA Shah NM Julius D Basbaum AI Trpv1 reporter mice reveal highly restricted brain distribution and functional expression in arteriolar smooth muscle cells J Neurosci 2011 31 5067 5077 10.1523/JNEUROSCI.6451-10.2011 21451044 PMC3087977

[B146] Dux M Santha P Jancso G Capsaicin-sensitive neurogenic sensory vasodilatation in the dura mater of the rat J Physiol 2003 552 859 867 10.1113/jphysiol.2003.050633 12949222 PMC2343470

[B147] Wang YX Wang J Wang C Liu J Shi LP Xu M Functional expression of transient receptor potential vanilloid-related channels in chronically hypoxic human pulmonary arterial smooth muscle cells J Membr Biol 2008 223 151 159 10.1007/s00232-008-9121-9 18787888

[B148] Hwang JT Park IJ Shin JI Lee YK Lee SK Baik HW Ha J Park OJ Genistein, EGCG, and capsaicin inhibit adipocyte differentiation process via activating AMP-activated protein kinase Biochem Biophys Res Commun 2005 338 694 699 10.1016/j.bbrc.2005.09.195 16236247

[B149] Hsu CL Yen GC Effects of capsaicin on induction of apoptosis and inhibition of adipogenesis in 3T3-L1 cells J Agric Food Chem 2007 55 1730 1736 10.1021/jf062912b 17295509

[B150] Zhang LL Yan Liu D Ma LQ Luo ZD Cao TB Zhong J Yan ZC Wang LJ Zhao ZG Zhu SJ Activation of transient receptor potential vanilloid type-1 channel prevents adipogenesis and obesity Circ Res 2007 100 1063 1070 10.1161/01.RES.0000262653.84850.8b 17347480

[B151] Ohnuki K Haramizu S Oki K Watanabe T Yazawa S Fushiki T Administration of capsiate, a non-pungent capsaicin analog, promotes energy metabolism and suppresses body fat accumulation in mice Biosci Biotechnol Biochem 2001 65 2735 2740 10.1271/bbb.65.2735 11826971

[B152] Kang JH Goto T Han IS Kawada T Kim YM Yu R Dietary capsaicin reduces obesity-induced insulin resistance and hepatic steatosis in obese mice fed a high-fat diet Obesity (Silver Spring) 2010 18 780 787 10.1038/oby.2009.301 19798065

[B153] Romanovsky AA Almeida MC Garami A Steiner AA Norman MH Morrison SF Nakamura K Burmeister JJ Nucci TB The transient receptor potential vanilloid-1 channel in thermoregulation: a thermosensor it is not Pharmacol Rev 2009 61 228 261 10.1124/pr.109.001263 19749171 PMC2763780

[B154] Caterina MJ Transient receptor potential ion channels as participants in thermosensation and thermoregulation Am J Physiol Regul Integr Comp Physiol 2007 292 R64 76 16973931 10.1152/ajpregu.00446.2006

[B155] Masamoto Y Kawabata F Fushiki T Intragastric administration of TRPV1, TRPV3, TRPM8, and TRPA1 agonists modulates autonomic thermoregulation in different manners in mice Biosci Biotechnol Biochem 2009 73 1021 1027 10.1271/bbb.80796 19420725

[B156] Kawabata F Inoue N Masamoto Y Matsumura S Kimura W Kadowaki M Higashi T Tominaga M Inoue K Fushiki T Non-pungent capsaicin analogs (capsinoids) increase metabolic rate and enhance thermogenesis via gastrointestinal TRPV1 in mice Biosci Biotechnol Biochem 2009 73 2690 2697 10.1271/bbb.90555 19966466

[B157] Toth DM Szoke E Bolcskei K Kvell K Bender B Bosze Z Szolcsanyi J Sandor Z Nociception, neurogenic inflammation and thermoregulation in TRPV1 knockdown transgenic mice Cell Mol Life Sci 2011 68 2589 2601 10.1007/s00018-010-0569-2 21069423 PMC11115187

[B158] Bodo E Biro T Telek A Czifra G Griger Z Toth BI Mescalchin A Ito T Bettermann A Kovacs L Paus R A hot new twist to hair biology: involvement of vanilloid receptor-1 (VR1/TRPV1) signaling in human hair growth control Am J Pathol 2005 166 985 998 10.1016/S0002-9440(10)62320-6 15793280 PMC1602392

[B159] White JP Urban L Nagy I TRPV1 function in health and disease Curr Pharm Biotechnol 2011 12 130 144 10.2174/138920111793937844 20932253

[B160] Cortright DN Krause JE Broom DC TRP channels and pain Biochim Biophys Acta 2007 1772 978 988 10.1016/j.bbadis.2007.03.003 17467247

[B161] Gunthorpe MJ Szallasi A Peripheral TRPV1 receptors as targets for drug development: new molecules and mechanisms Curr Pharm Des 2008 14 32 41 10.2174/138161208783330754 18220816

[B162] Toth A Boczan J Kedei N Lizanecz E Bagi Z Papp Z Edes I Csiba L Blumberg PM Expression and distribution of vanilloid receptor 1 (TRPV1) in the adult rat brain Brain Res Mol Brain Res 2005 135 162 168 10.1016/j.molbrainres.2004.12.003 15857679

[B163] Yiangou Y Facer P Dyer NH Chan CL Knowles C Williams NS Anand P Vanilloid receptor 1 immunoreactivity in inflamed human bowel Lancet 2001 357 1338 1339 10.1016/S0140-6736(00)04503-7 11343743

[B164] Chan CL Facer P Davis JB Smith GD Egerton J Bountra C Williams NS Anand P Sensory fibres expressing capsaicin receptor TRPV1 in patients with rectal hypersensitivity and faecal urgency Lancet 2003 361 385 391 10.1016/S0140-6736(03)12392-6 12573376

[B165] Welch JM Simon SA Reinhart PH The activation mechanism of rat vanilloid receptor 1 by capsaicin involves the pore domain and differs from the activation by either acid or heat Proc Natl Acad Sci U S A 2000 97 13889 13894 10.1073/pnas.230146497 11095706 PMC17671

[B166] Kuzhikandathil EV Wang H Szabo T Morozova N Blumberg PM Oxford GS Functional analysis of capsaicin receptor (vanilloid receptor subtype 1) multimerization and agonist responsiveness using a dominant negative mutation J Neurosci 2001 21 8697 8706 11698581 10.1523/JNEUROSCI.21-22-08697.2001 PMC6762288

[B167] Grandl J Kim SE Uzzell V Bursulaya B Petrus M Bandell M Patapoutian A Temperature-induced opening of TRPV1 ion channel is stabilized by the pore domain Nat Neurosci 2010 13 708 714 10.1038/nn.2552 20414199 PMC2876202

[B168] Jordt SE Julius D Molecular basis for species-specific sensitivity to "hot" chili peppers Cell 2002 108 421 430 10.1016/S0092-8674(02)00637-2 11853675

[B169] Ho KW Ward NJ Calkins DJ TRPV1: a stress response protein in the central nervous system Am J Neurodegener Dis 2012 1 1 14 22737633 PMC3560445

[B170] Chou MZ Mtui T Gao YD Kohler M Middleton RE Resiniferatoxin binds to the capsaicin receptor (TRPV1) near the extracellular side of the S4 transmembrane domain Biochemistry 2004 43 2501 2511 10.1021/bi035981h 14992587

[B171] Gavva NR Klionsky L Qu Y Shi L Tamir R Edenson S Zhang TJ Viswanadhan VN Toth A Pearce LV Molecular determinants of vanilloid sensitivity in TRPV1 J Biol Chem 2004 279 20283 20295 10.1074/jbc.M312577200 14996838

[B172] Fernandez-Ballester G Ferrer-Montiel A Molecular modeling of the full-length human TRPV1 channel in closed and desensitized states J Membr Biol 2008 223 161 172 10.1007/s00232-008-9123-7 18791833

[B173] Johnson DM Garrett EM Rutter R Bonnert TP Gao YD Middleton RE Sutton KG Functional mapping of the transient receptor potential vanilloid 1 intracellular binding site Mol Pharmacol 2006 70 1005 1012 10.1124/mol.106.023945 16763090

[B174] Numazaki M Tominaga T Toyooka H Tominaga M Direct phosphorylation of capsaicin receptor VR1 by protein kinase Cepsilon and identification of two target serine residues J Biol Chem 2002 277 13375 13378 10.1074/jbc.C200104200 11884385

[B175] Susankova K Ettrich R Vyklicky L Teisinger J Vlachova V Contribution of the putative inner-pore region to the gating of the transient receptor potential vanilloid subtype 1 channel (TRPV1) J Neurosci 2007 27 7578 7585 10.1523/JNEUROSCI.1956-07.2007 17626219 PMC6672601

[B176] Mohapatra DP Wang SY Wang GK Nau C A tyrosine residue in TM6 of the Vanilloid Receptor TRPV1 involved in desensitization and calcium permeability of capsaicin-activated currents Mol Cell Neurosci 2003 23 314 324 10.1016/S1044-7431(03)00054-X 12812762

[B177] Myers BR Bohlen CJ Julius D A yeast genetic screen reveals a critical role for the pore helix domain in TRP channel gating Neuron 2008 58 362 373 10.1016/j.neuron.2008.04.012 18466747 PMC2422846

[B178] Salazar H Jara-Oseguera A Hernandez-Garcia E Llorente I Arias O II Soriano-Garcia M Islas LD Rosenbaum T Structural determinants of gating in the TRPV1 channel Nat Struct Mol Biol 2009 16 704 710 10.1038/nsmb.1633 19561608

[B179] Boukalova S Marsakova L Teisinger J Vlachova V Conserved residues within the putative S4-S5 region serve distinct functions among thermosensitive vanilloid transient receptor potential (TRPV) channels J Biol Chem 2010 285 41455 41462 10.1074/jbc.M110.145466 21044960 PMC3009871

[B180] Lee JH Lee Y Ryu H Kang DW Lee J Lazar J Pearce LV Pavlyukovets VA Blumberg PM Choi S Structural insights into transient receptor potential vanilloid type 1 (TRPV1) from homology modeling, flexible docking, and mutational studies J Comput Aided Mol Des 2011 25 317 327 10.1007/s10822-011-9421-5 21448716 PMC3420359

[B181] Bautista DM Movahed P Hinman A Axelsson HE Sterner O Hogestatt ED Julius D Jordt SE Zygmunt PM Pungent products from garlic activate the sensory ion channel TRPA1 Proc Natl Acad Sci U S A 2005 102 12248 12252 10.1073/pnas.0505356102 16103371 PMC1189336

[B182] Hinman A Chuang HH Bautista DM Julius D TRP channel activation by reversible covalent modification Proc Natl Acad Sci U S A 2006 103 19564 19568 10.1073/pnas.0609598103 17164327 PMC1748265

[B183] Bandell M Story GM Hwang SW Viswanath V Eid SR Petrus MJ Earley TJ Patapoutian A Noxious cold ion channel TRPA1 is activated by pungent compounds and bradykinin Neuron 2004 41 849 857 10.1016/S0896-6273(04)00150-3 15046718

[B184] Macpherson LJ Dubin AE Evans MJ Marr F Schultz PG Cravatt BF Patapoutian A Noxious compounds activate TRPA1 ion channels through covalent modification of cysteines Nature 2007 445 541 545 10.1038/nature05544 17237762

[B185] Rosenbaum T Castanares DT Lopez-Valdes HE Hiriart M Nerve growth factor increases L-type calcium current in pancreatic beta cells in culture J Membr Biol 2002 186 177 184 10.1007/s00232-001-0143-9 12148844

[B186] Salazar H Llorente I Jara-Oseguera A Garcia-Villegas R Munari M Gordon SE Islas LD Rosenbaum T A single N-terminal cysteine in TRPV1 determines activation by pungent compounds from onion and garlic Nat Neurosci 2008 11 255 261 10.1038/nn2056 18297068 PMC4370189

[B187] Latorre R Zaelzer C Brauchi S Structure-functional intimacies of transient receptor potential channels Q Rev Biophys 2009 42 201 246 10.1017/S0033583509990072 20025796

[B188] Brauchi S Orta G Salazar M Rosenmann E Latorre R A hot-sensing cold receptor: C-terminal domain determines thermosensation in transient receptor potential channels J Neurosci 2006 26 4835 4840 10.1523/JNEUROSCI.5080-05.2006 16672657 PMC6674176

[B189] Yao J Liu B Qin F Modular thermal sensors in temperature-gated transient receptor potential (TRP) channels Proc Natl Acad Sci U S A 2011 108 11109 11114 10.1073/pnas.1105196108 21690353 PMC3131340

[B190] Cui Y Yang F Cao X Yarov-Yarovoy V Wang K Zheng J Selective disruption of high sensitivity heat activation but not capsaicin activation of TRPV1 channels by pore turret mutations J Gen Physiol 2012 139 273 283 10.1085/jgp.201110724 22412190 PMC3315147

[B191] Brauchi S Orta G Mascayano C Salazar M Raddatz N Urbina H Rosenmann E Gonzalez-Nilo F Latorre R Dissection of the components for PIP2 activation and thermosensation in TRP channels Proc Natl Acad Sci U S A 2007 104 10246 10251 10.1073/pnas.0703420104 17548815 PMC1891241

[B192] Ryu S Liu B Yao J Fu Q Qin F Uncoupling proton activation of vanilloid receptor TRPV1 J Neurosci 2007 27 12797 12807 10.1523/JNEUROSCI.2324-07.2007 18032651 PMC6673297

[B193] Sutton KG Garrett EM Rutter AR Bonnert TP Jarolimek W Seabrook GR Functional characterisation of the S512Y mutant vanilloid human TRPV1 receptor Br J Pharmacol 2005 146 702 711 10.1038/sj.bjp.0706356 16100528 PMC1751200

[B194] Grimm C Aneiros E de Groot M Dissecting TRPV1: lessons to be learned? Channels (Austin) 2011 5 201 204 10.4161/chan.5.3.16291 21654202

[B195] Aneiros E Cao L Papakosta M Stevens EB Phillips S Grimm C The biophysical and molecular basis of TRPV1 proton gating EMBO J 2011 30 994 1002 10.1038/emboj.2011.19 21285946 PMC3061026

[B196] Voets T Owsianik G Janssens A Talavera K Nilius B TRPM8 voltage sensor mutants reveal a mechanism for integrating thermal and chemical stimuli Nat Chem Biol 2007 3 174 182 10.1038/nchembio862 17293875

[B197] Garcia-Martinez C Morenilla-Palao C Planells-Cases R Merino JM Ferrer-Montiel A Identification of an aspartic residue in the P-loop of the vanilloid receptor that modulates pore properties J Biol Chem 2000 275 32552 32558 10931826 10.1074/jbc.M002391200

[B198] Bohlen CJ Priel A Zhou S King D Siemens J Julius D A bivalent tarantula toxin activates the capsaicin receptor, TRPV1, by targeting the outer pore domain Cell 2010 141 834 845 10.1016/j.cell.2010.03.052 20510930 PMC2905675

[B199] Siemens J Zhou S Piskorowski R Nikai T Lumpkin EA Basbaum AI King D Julius D Spider toxins activate the capsaicin receptor to produce inflammatory pain Nature 2006 444 208 212 10.1038/nature05285 17093448

[B200] Kitaguchi T Swartz KJ An inhibitor of TRPV1 channels isolated from funnel Web spider venom Biochemistry 2005 44 15544 15549 10.1021/bi051494l 16300403

[B201] Grycova L Lansky Z Friedlova E Obsilova V Janouskova H Obsil T Teisinger J Ionic interactions are essential for TRPV1 C-terminus binding to calmodulin Biochem Biophys Res Commun 2008 375 680 683 10.1016/j.bbrc.2008.08.094 18755153

[B202] Grycova L Lansky Z Friedlova E Vlachova V Kubala M Obsilova V Obsil T Teisinger J ATP binding site on the C-terminus of the vanilloid receptor Arch Biochem Biophys 2007 465 389 398 10.1016/j.abb.2007.06.035 17706589

[B203] Kwon Y Hofmann T Montell C Integration of phosphoinositide- and calmodulin-mediated regulation of TRPC6 Mol Cell 2007 25 491 503 10.1016/j.molcel.2007.01.021 17317623 PMC1855209

[B204] Zhu MX Multiple roles of calmodulin and other Ca(2+)-binding proteins in the functional regulation of TRP channels Pflugers Arch 2005 451 105 115 10.1007/s00424-005-1427-1 15924238

[B205] Kwak J Wang MH Hwang SW Kim TY Lee SY Oh U Intracellular ATP increases capsaicin-activated channel activity by interacting with nucleotide-binding domains J Neurosci 2000 20 8298 8304 11069936 10.1523/JNEUROSCI.20-22-08298.2000 PMC6773187

[B206] Grycova L Holendova B Bumba L Bily J Jirku M Lansky Z Teisinger J Integrative binding sites within intracellular termini of TRPV1 receptor PLoS ONE 2012 7 e48437 10.1371/journal.pone.0048437 23119017 PMC3485206

[B207] Tousova K Susankova K Teisinger J Vyklicky L Vlachova V Oxidizing reagent copper-o-phenanthroline is an open channel blocker of the vanilloid receptor TRPV1 Neuropharmacology 2004 47 273 285 10.1016/j.neuropharm.2004.04.001 15223306

[B208] Picazo-Juarez G Romero-Suarez S Nieto-Posadas A Llorente I Jara-Oseguera A Briggs M McIntosh TJ Simon SA Ladron-de-Guevara E Islas LD Rosenbaum T Identification of a binding motif in the S5 helix that confers cholesterol sensitivity to the TRPV1 ion channel J Biol Chem 2011 286 24966 24976 10.1074/jbc.M111.237537 21555515 PMC3137070

[B209] Numazaki M Tominaga M Nociception and TRP Channels Curr Drug Targets CNS Neurol Disord 2004 3 479 485 10.2174/1568007043336789 15578965

[B210] Mohapatra DP Nau C Desensitization of capsaicin-activated currents in the vanilloid receptor TRPV1 is decreased by the cyclic AMP-dependent protein kinase pathway J Biol Chem 2003 278 50080 50090 10.1074/jbc.M306619200 14506258

[B211] Zhang X Huang J McNaughton PA NGF rapidly increases membrane expression of TRPV1 heat-gated ion channels EMBO J 2005 24 4211 4223 10.1038/sj.emboj.7600893 16319926 PMC1356334

[B212] Wirkner K Hognestad H Jahnel R Hucho F Illes P Characterization of rat transient receptor potential vanilloid 1 receptors lacking the N-glycosylation site N604 Neuroreport 2005 16 997 1001 10.1097/00001756-200506210-00023 15931076

[B213] Pecze L Winter Z Josvay K Otvos F Kolozsi C Vizler C Budai D Letoha T Dombi G Szakonyi G Olah Z Divalent heavy metal cations block the TRPV1 Ca(2+) channel Biol Trace Elem Res 2012 151 451 461 23264033 10.1007/s12011-012-9570-y PMC3566393

[B214] Garcia-Sanz N Fernandez-Carvajal A Morenilla-Palao C Planells-Cases R Fajardo-Sanchez E Fernandez-Ballester G Ferrer-Montiel A Identification of a tetramerization domain in the C terminus of the vanilloid receptor J Neurosci 2004 24 5307 5314 10.1523/JNEUROSCI.0202-04.2004 15190102 PMC6729306

