# Supplementary figures and images for: Functionally important amino acid residues in the transient receptor potential vanilloid 1 (TRPV1) ion channel – an overview of the current mutational data
Source: Mol Pain. 2013 Jun 22;9:30. doi: 10.1186/1744-8069-9-30 (PMC3707783; doi:10.1186/1744-8069-9-30)

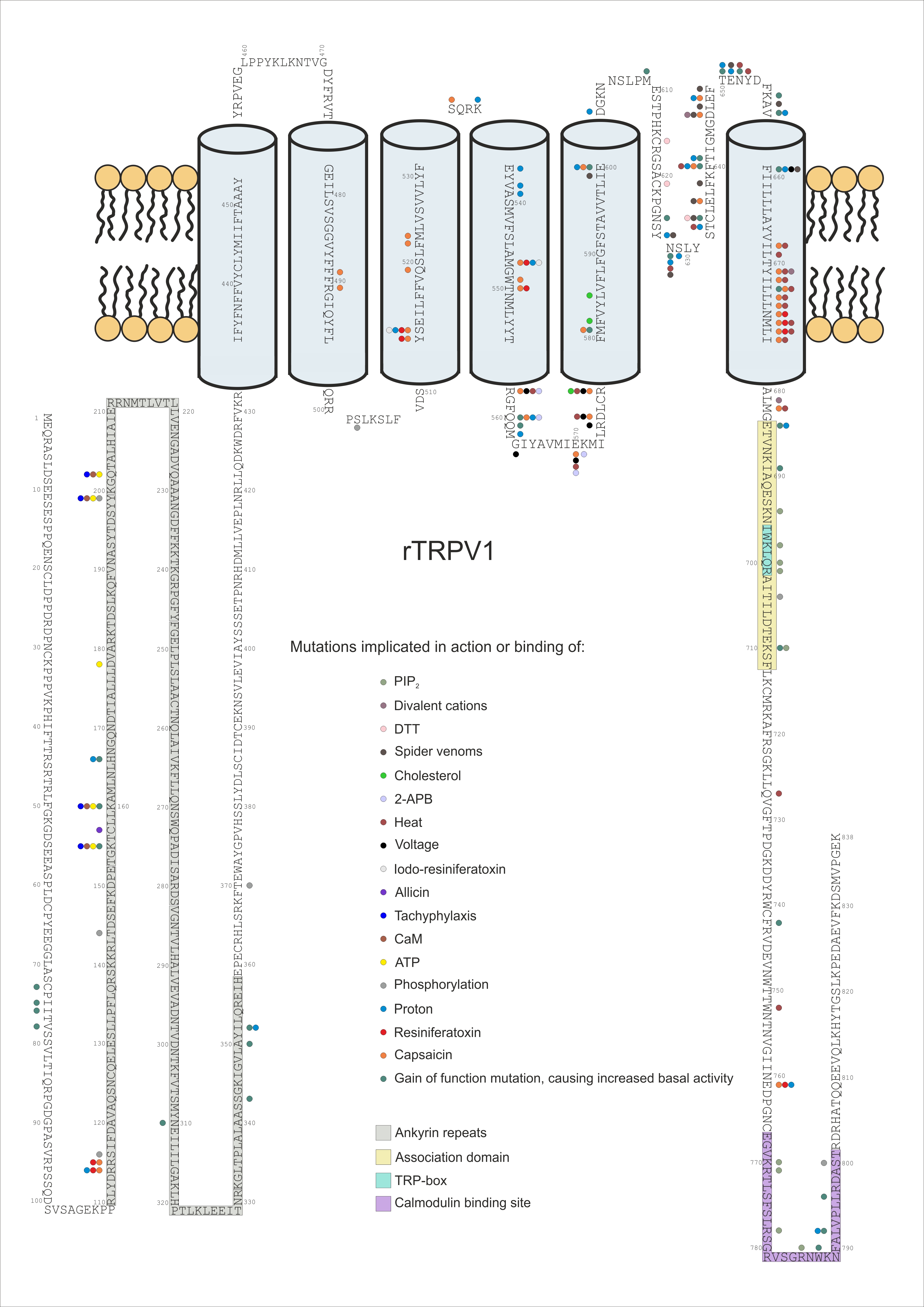

Supplement: Additional file 1 — Summary of the mutated sites of rTRPV1. [file 1744-8069-9-30-S1.jpeg]
